# Repeated-Dose Toxicity of Lauric Acid and Its Preventive Effect Against Tracheal Hyper-Responsiveness in Wistar Rats with Possible *In Silico* Molecular Targets

**DOI:** 10.3390/ph18020221

**Published:** 2025-02-06

**Authors:** Indyra Alencar Duarte Figueiredo, Alissa Maria de Oliveira Martins, Alexya Mikelle Teixeira Cavalcanti, Jayne Muniz Fernandes, Ludmila Emilly da Silva Gomes, Mateus Mendes Vieira, Gabriel Nunes Machado de Oliveira, Isabela Motta Felício, Lucas Nóbrega de Oliveira, Igor Gabriel da Silva Ramalho, Natália Ferreira de Sousa, Luciana Scotti, Marcus Tullius Scotti, José Luiz de Brito Alves, Margareth de Fátima Formiga Melo Diniz, Daniele Idalino Janebro Ximenes, Luiz Henrique César Vasconcelos, Fabiana de Andrade Cavalcante

**Affiliations:** 1 Laboratório de Farmacologia Funcional Prof. George Thomas, Instituto de Pesquisa em Fármacos e Medicamentos, Universidade Federal da Paraíba, João Pessoa 58051-900, PB, Brazil; alissaoliveira@ltf.ufpb.br (A.M.d.O.M.); alexyacavalcanti08@gmail.com (A.M.T.C.); jaynemunizf@gmail.com (J.M.F.); ludmilaesgomes@gmail.com (L.E.d.S.G.); mateusmvd89@gmail.com (M.M.V.); gabrielsoad619@gmail.com (G.N.M.d.O.); isabela_motta@ltf.ufpb.br (I.M.F.); lucasnobrega@ltf.ufpb.br (L.N.d.O.); igorgabriel0809@gmail.com (I.G.d.S.R.); nataliafsousa@ltf.ufpb.br (N.F.d.S.); fabianacavalcante@ltf.ufpb.br (F.d.A.C.); 2 Programa de Pós-Graduação em Produtos Naturais e Sintéticos Bioativos, Centro de Ciências da Saúde, Universidade Federal da Paraíba, João Pessoa 58051-900, PB, Brazil; luciana.scotti@gmail.com (L.S.); mtscotti@gmail.com (M.T.S.); margarethdiniz.ufpb@gmail.com (M.d.F.F.M.D.); 3 Departamento de Química, Centro de Ciências Exatas e da Natureza, Universidade Federal da Paraíba, João Pessoa 58051-900, PB, Brazil; 4 Departamento de Nutrição, Centro de Ciências da Saúde, Universidade Federal da Paraíba, João Pessoa 58051-900, PB, Brazil; jose.luiz@academico.ufpb.br; 5 Departamento de Ciências Farmacêuticas, Centro de Ciências da Saúde, Universidade Federal da Paraíba, Cidade Universitária, João Pessoa 58051-900, PB, Brazil; dijanebro@yahoo.com.br; 6 Departamento de Ciências Biomédicas, Centro de Ciências da Saúde, Universidade Federal da Paraíba, Cidade Universitária, João Pessoa 58051-900, PB, Brazil

**Keywords:** dodecanoic acid, allergic inflammation, pulmonary ventilation, tracheal reactivity, ovalbumin, molecular docking

## Abstract

Background/Objectives: Lauric acid (LA), a medium-chain fatty acid, is a promising drug for asthma treatment. This study evaluated the toxicity of repeated doses and the effect of LA on pulmonary ventilation and tracheal reactivity in asthmatic Wistar rats and identified possible molecular targets of LA action *in silico*. Methods: The rats were divided into control (CG) and LA-treated groups at 100 mg/kg (AL100G) for toxicity analysis. Pulmonary ventilation and tracheal reactivity were assessed in the control (CG), asthmatic (AG), asthmatic treated with LA at 25, 50, or 100 mg/kg (AAL25G, AAL50G, and AAL100G), and dexamethasone-treated groups (ADEXAG). Results: The results showed that LA at a dose of 100 mg/kg did not cause death or toxicity. A pulmonary ventilation analysis indicated that AG had reduced minute volume, which was prevented in AAL25G. LA at all doses prevented carbachol-induced tracheal hyper-responsiveness and reduced the relaxing effect of aminophylline, as observed in AG. An *in silico* analysis revealed that LA had a good affinity for nine proteins (β_2_-adrenergic receptor, Ca_V_, BK_Ca_, K_ATP_, adenylyl cyclase, PKG, eNOS, iNOS, and COX-2). Conclusions: LA at 100 mg/kg has low toxicity, prevents hyper-responsiveness in an asthma model in rats, and acts as a multitarget compound with a good affinity for proteins related to airway hyper-responsiveness.

## 1. Introduction

Asthma is a chronic inflammatory disease characterized by airway hyper-responsiveness, varying degrees of airflow obstruction, and lung inflammation. It affects more than 300 million individuals worldwide, with prevalence rates ranging from 1 to 29% of the population in different countries [1].

Therefore, studies aimed at gaining a better understanding of the pathophysiological mechanisms of asthma, as well as the search for more efficient and effective additional treatments, are necessary [2]. In this context, natural products stand out as a starting point for discovery, as they contain compounds with structural novelty and complexity that generate various biological activities [3,4], such as vegetable oils, including coconut oil obtained from the species *Cocos nucifera* L.

There are reports that the inhalation of virgin coconut oil (VCO) in an ovalbumin-induced allergic asthma model in rabbits reversed the infiltration of inflammatory cells, tissue remodeling, and the number of goblet and proliferative cells [5]. Furthermore, VCO supplementation reversed peribronchial inflammatory infiltration, epithelial hyperplasia, smooth muscle thickening, and tracheal hyper-reactivity in an ovalbumin-induced asthma model in guinea pigs through antioxidant mechanisms and the nitric oxide (NO) pathway [6]. This natural product is an essential source of fatty acids [7,8]. Based on this, it was hypothesized that lauric acid could be the compound responsible for the reversal activities of the changes promoted by the asthma induction models exerted by virgin coconut oil.

Among the main compounds found in coconut oil, lauric acid (LA) or dodecanoic acid is highlighted, a medium-chain saturated fatty acid that contains 12 carbon atoms [9,10], accounting for 45.1–53.2% of the total fatty acids [11], and is associated with some of the pharmacological activities promoted by coconut oil [12,13].

Lauric acid (LA) has been reported to have several beneficial effects, such as the prevention of benign prostatic hyperplasia [14], antidiabetic [15], neuroprotective [16,17], neuronal maturation [18], antimicrobial [19,20], antitumor effects [21,22], anti-obesity [23], reduction in pro-atherogenic factors [24], reduction in hepatometabolic complications in non-alcoholic fatty liver disease [25], and reduction in liver inflammation [26], in addition to exhibiting antihypertensive and vasorelaxant activity [12].

Therefore, this study aimed to evaluate the safety of using lauric acid in repeated doses and investigate its effects in preventing alterations in pulmonary ventilation and tracheal hyper-responsiveness in an ovalbumin-induced asthma model in Wistar rats. Additionally, *in silico* molecular docking studies were conducted to evaluate the potential molecular interaction targets related to airway smooth muscle reactivity.

## 2. Results and Discussion

### 2.1. Repeated-Dose Toxicity and Behavioral Pharmacological Screening of Lauric Acid

To ensure the safety of using lauric acid in the other *in vivo* experimental protocols, a repeated-dose toxicity test was conducted by administering 100 mg/kg of lauric acid daily for 28 days, followed by an observation period of 14 days without the drug to assess the persistence of any potential toxic effects after treatment. No animal deaths were observed at the end of the 42-day observation period.

Additionally, a behavioral, pharmacological screening test was conducted on the first day following a single dose administration of 100 mg/kg of lauric acid to quantify behavioral and physiological parameters regarding consciousness, motor coordination, muscle tone, and reflexes related to changes in the central and peripheral nervous systems [27]. These parameters were observed within 30 min and every hour for 4 h after administration.

The control group animals showed no clinical signs during the initial 4 h evaluation. In contrast, one male rat in the GAL100 group exhibited increased analgesia during the first 30 min; however, this condition reversed in the subsequent hours of observation.

Overall, lauric acid at a dose of 100 mg/kg did not produce toxic effects on the behavioral parameters observed in the days following the first administration, nor was there any lethality throughout the treatment protocol, ensuring the safety of the oral administration of this drug in the other *in vivo* experimental protocols.

### 2.2. Effect of Lauric Acid on Weight Gain, Food and Water Intake, and Organ Weight

Signs of systemic toxicity can be defined by a reduction in the body mass of experimental animals due to decreased food consumption, serving as indicators of intense pain, distress, suffering, or imminent death, and thus address the results characterized as exceeding the threshold of suffering [28,29]. The animals were weighed before administering lauric acid and periodically during the 28-day treatment, extending to the end of the observation period, totaling 42 days. When assessing the weight evolution of the group treated with lauric acid at a dose of 100 mg/kg, no significant changes in weight gain were observed when comparing females (47.0 ± 2.9 g) and males (111.6 ± 9.2 g) with the control group animals (48.0 ± 4.2 g and 113.8 ± 8.2 g, respectively) (Table 1).

Concurrently, the average weekly food and water consumption for all the animals was monitored (Table 2). The females in the AL100 group exhibited a weekly food (162.9 ± 22.3 g) and water intake (90.2 ± 5.5 mL) that did not differ from the control group (CG) (178.2 ± 24.8 g and 103.2 ± 5.6 mL, respectively). Similarly, the males in the AL100G group also showed no differences in food (236.6 ± 20.3 g) and water intake (145.6 ± 10.4 mL) when compared to the control group (254.8 ± 16.9 g; 180.3 ± 12.5 mL, respectively).

At the end of the experimental period, the animals were euthanized, and the internal organs were observed for the macromorphological evaluation and identification of possible lesions, followed by their removal and weighing, considering that organ weight is one of the most sensitive indicators of the potential toxic effects of the test drug. Variations in organ weight are generally accompanied by differences in body weight among the treatment groups, which complicates the interpretation of the results related to organ weight [30].

Thus, the relative weights of the organs were determined across different groups, with no significant differences observed between the experimental groups (Table 3). Furthermore, no macromorphological changes were evident, indicating that the daily consumption of lauric acid at a dose of 100 mg/kg did not induce macroscopic morphological alterations or changes in the weight of the evaluated organs.

The results obtained are consistent with those described by Khan et al. (2020), where an acute toxicity protocol was performed with lauric acid in female Sprague Dawley rats, with a minimum dose of 300 mg/kg and a maximum of 2000 mg/kg [31], indicating that lauric acid up to a dose of 100 mg/kg can be considered a safe drug for use in *in vivo* experimental protocols in animals as it did not present a toxic profile at the tested doses.

### 2.3. Effect of Lauric Acid on Hematological Parameters

The analysis of hematological parameters is recommended in toxicological studies, as the hematopoietic system is most affected by therapeutic or toxic agents [32]. It was observed that no hematological parameters were altered in males between the experimental groups (Table 4).

In females, although some parameters such as mean corpuscular hemoglobin (17.5 ± 0.3 and 18.6 ± 0.1%), segmented neutrophils (23.0 ± 1.7 and 27.8 ± 0.6%), lymphocytes (74.6 ± 1.9 and 69.8 ± 0.6%), and platelets (1069.0 ± 65.6 and 1075.0 ± 53.9 103/mm^3^) were changed in the AL100G group compared to the control group (CG), respectively, all the alterations are within the reference value limits stipulated [33] and are related to the expected homeostatic variation among individuals (Table 5). Consequently, these effects are considered incidental, unrelated to the administration of lauric acid, and therefore, without toxicological relevance.

These results also agree with those observed by Khan et al. (2020), in which lauric acid, in the acute toxicity protocol at doses of 300 and 2000 mg/kg, did not present changes in hematological parameters in Sprague Dawley rats [31].

### 2.4. Effect of Lauric Acid on Biochemical Parameters

Evaluations of hepatic and renal function in biochemical analyses are essential for monitoring the toxicity or dysfunction of specific organs caused by toxic agents, as the toxicity of substances often manifests as hepatic and/or renal irregularities due to the involvement of these organs in metabolism [34].

Exposure to toxic compounds can lead to hepatotoxicity, resulting in cirrhosis, fibrosis, and liver damage, which can be assessed by measuring glucose and cholesterol levels, given that the liver is a central organ in regulating the homeostasis of these macromolecules [35,36]. Additionally, the quantification of liver enzymes ALT (alanine aminotransferase) and AST (aspartate aminotransferase), which are markers of hepatocyte injury, is necessary [37]. Urea and uric acid are byproducts of protein metabolism that the kidneys remove, making them essential for evaluating renal function [38].

It was observed that the males in the AL100G group showed an increase in cholesterol (85.4 ± 5.3 mg/dL) compared to the CG (66.4 ± 2.3 mg/dL); however, these values are within the reference limits [33]. This result is consistent with what has been reported in the literature regarding lauric acid administration. Denke (2006) concluded that all the saturated fatty acids with chain lengths of 8 to 16 carbon atoms resulted in increased cholesterol levels [39]. Furthermore, it was demonstrated that lauric acid caused an increase in total cholesterol, with a more significant proportion of the increase in high-density lipoprotein (HDL), which is involved in the reverse transport of cholesterol from peripheral organs to the liver, thus contributing to a lower risk of atherosclerotic cardiovascular disease [40,41,42,43,44,45,46,47,48,49]. No changes were observed regarding the other biochemical parameters (Table 6).

The females in the AL100G group showed a reduction in serum urea levels (34.4 ± 2.7 U/L) compared to those in the CG (42.4 ± 1.2 U/L). However, despite the difference between the groups, this parameter remains within the reference values [33]. In the quantification of urea, no changes were observed between the experimental groups of males (Table 7). Similar results were also observed by Khan et al. (2020), where lauric acid in an acute toxicity protocol at doses of 300 and 2000 mg/kg did not show alterations in biochemical parameters in Sprague Dawley rats [31].

Thus, according to the results obtained from the biochemical markers related to liver and kidney function, it can be concluded that the toxicological evaluation with lauric acid at a dose of 100 mg/kg did not produce liver and kidney damage, making it a safe drug for oral administration at the dose tested.

### 2.5. Effect of Lauric Acid on Airway Hyper-Responsiveness

#### 2.5.1. Respiratory Parameters

The pulmonary ventilation test is an essential component in evaluating the outcomes of experimental models of respiratory diseases in rodents. It can assess the physiological parameters of underlying lung disease [42].

Whole-body plethysmography chambers are commonly used to assess ventilatory function and directly monitor changes in lung volume or airflow generated by thoracic movements [43]. During inspiration, the incoming air is heated and humidified within the lungs, increasing water vapor pressure and causing the thermal expansion of the gas. This effect causes a change in air volume, increasing pressure within the plethysmography chamber. The opposite occurs during expiration, creating a respiratory waveform in the animal [44].

One of the respiratory parameters evaluated was tidal volume (VT), which corresponds to the air inhaled or exhaled during a normal breath at rest [45]. VT is an essential physiological parameter to ensure efficient pulmonary ventilation and gas homeostasis in the body. During inspiration, the oxygen in atmospheric air is transported to the lungs, crossing the alveolar-capillary barrier by diffusion and entering the arterial circulation. Simultaneously, carbon dioxide, continuously produced as a byproduct of cellular metabolism, is transferred from the venous blood to the pulmonary alveoli. The expiration process eliminates carbon dioxide, preventing its accumulation in the body. Thus, the volume of air mobilized in each respiratory cycle called tidal volume is crucial to maintaining balanced oxygen and carbon dioxide levels in arterial and venous blood, supporting the body’s metabolic demands [46].

**Figure 1:**
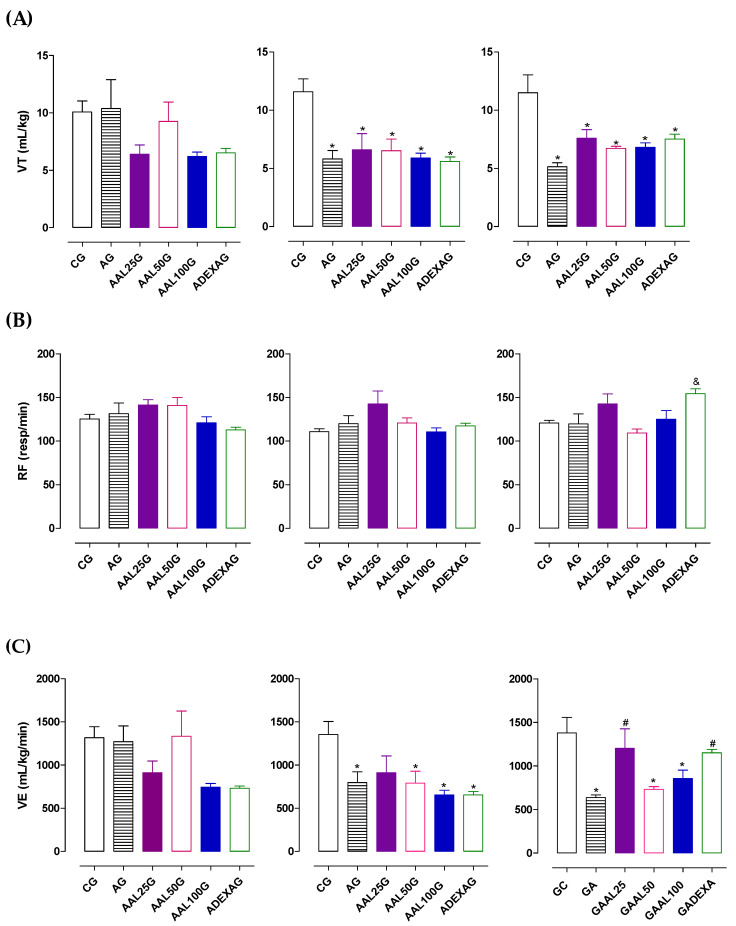
Tidal volume (**A**), respiratory rate (**B**), and minute volume (**C**) of the animals from CG, AG, AAL25G, AAL50G, AAL100G, and ADEXAG on days 1, 12, and 21 of asthma induction. The symbols and vertical bars represent the mean and S.E.M., respectively. ANOVA one-way followed by Tukey’s post hoc test (n = 6). * *p* < 0.05 (CG vs. AG, AAL25G, AAL50G, AAL100G, and ADEXAG); ^#^ *p* < 0.05 (AG vs. AAL25G, AAL50G, AAL100G, and ADEXAG); ^&^ *p* < 0.05 (AAL50G vs. AG, AAL25G, AAL100G, and ADEXAG).

The animals in the AG (10.4 ± 2.5 mL/kg), AAL25G (6.4 ± 0.8 mL/kg), AAL50G (9.3 ± 1.7 mL/kg), AAL100G (6.2 ± 0.4 mL/kg), and ADEXAG (6.5 ± 0.4 mL/kg) groups did not show changes in the tidal volume (VT) compared to the control group (10.1 ± 0.9 mL/kg) on the first day of asthma induction (Figure 1). However, on days 12 (5.8 ± 0.7; 6.6 ± 1.4; 6.5 ± 1.1; 5.9 ± 0.4; and 5.6 ± 0.4 mL/kg, respectively) and 21 (5.1 ± 0.3; 7.6 ± 0.7; 6.7 ± 0.2; 6.8 ± 0.4; and 7.5 ± 0.4 mL/kg, respectively), all the groups exhibited a reduction in VT when compared to the CG (11.6 ± 1.1 and 11.5 ± 1.5 mL/kg) (n = 6). In other studies of allergic asthma induced by ovalbumin in rats, a reduction in tidal volume was also observed in the asthmatic group compared to the control group [47,48,49].

The respiratory frequency (RF) is represented by the total number of respiratory cycles per minute (inspiration and expiration). It is a crucial physiological parameter the central nervous system regulates in response to chemical stimuli, such as carbon dioxide levels, oxygen, and pH in the blood [50]. RF was not altered on days 1, 12, or 21 (Figure 1) in the AG (131.3 ± 12.2; 120.0 ± 9.1; and 119.6 ± 11.4 breaths/min, respectively), AAL25G (141.4 ± 6.0; 142.6 ± 14.8; and 142.5 ± 11.5 breaths/min, respectively), AAL50G (140.9 ± 9.1; 120.6 ± 6.0; and 109.1 ± 4.8 breaths/min, respectively), AAL100G (120.9 ± 6.8; 110.6 ± 4.4; and 124.8 ± 10.0 breaths/min, respectively), and ADEXAG (112.9 ± 3.0; 117.3 ± 3.1; and 154.3 ± 5.4 breaths/min, respectively) compared to the CG (125.2 ± 5.3; 110.8 ± 3.2; and 120.8 ± 2.8 breaths/min, respectively) (n = 6).

Other studies in an ovalbumin-induced allergic asthma model in rodents reported an increase in respiratory frequency in the asthmatic group compared to the control group [51,52,53,54,55].

Studies by Mukherjee et al. (2017) and Parlar and Arslan (2020) reported that in a rat model of allergic asthma induced by ovalbumin, a reduction in ventilation minute (VE) can be observed in the asthmatic group compared to the control group [47,48]. This parameter refers to the product of the respiratory frequency and the tidal volume, representing the total volume of air inhaled in one minute [50].

Similarly, ventilation minute (n = 6) was not altered on day 1 in the AG (1270 ± 182.0 mL/kg/min), AAL25G (910.7 ± 136.1 mL/kg/min), AAL50G (1333 ± 291.4 mL/kg/min), AAL100G (744.0 ± 42.2 mL/kg/min), and ADEXAG (731.1 ± 26.2 mL/kg/min) groups. On days 12 and 21, VE was reduced in AG (797.1 ± 123.4 and 636.2 ± 31.5 mL/kg/min, respectively) when compared with CG (1353 ± 151.3 and 1379 ± 179.5 mL/kg/min, respectively).

The animals of AAL50G and AAL100G showed a reduction in VE on days 12 (790.6 ± 137.4 and 651.7 ± 55.6 mL/kg/min, respectively) and 21 (731.7 ± 31.1 and 855.6 ± 97.0 mL/kg/min, respectively) when compared with the CG. AAL25G did not show any change in this parameter on days 1 and 12 (910.7 ± 136.1; 911.6 ± 195.1 mL/kg/min) when compared with the CG. On the other hand, AAL25G prevented the reduction in minute volume observed in GA on day 21 (1203 ± 224.3 mL/kg/min) of asthma induction. Minute volume was reduced in ADEXAG on day 12 (653.1 ± 39.1 mL/kg/min) when compared with CG, and on day 21, treatment with dexamethasone reversed the reduction in ventilation minute (1151 ± 39.9 mL/kg/min) presented in AG (Figure 1).

Thus, these results show an alteration in respiratory mechanics and suggest a reduction in airflow, characteristic of increased airway resistance and hyper-responsiveness in AG, which was prevented by treatment with lauric acid at 25 mg/kg.

#### 2.5.2. Investigation of Contractile Reactivity to Ovalbumin—Schultz–Dale Reaction

The establishment of the asthma model in Wistar rats can also be demonstrated in *in vitro* protocols through the Schultz–Dale reaction, a standard technique to confirm a model of local anaphylactic response. This reaction is characterized by the release of contractile mediators, primarily histamine, and contractile factors derived from the epithelium, such as prostaglandins and leukotrienes [56], in antigenically sensitized animals upon re-exposure to OVA, culminating in the constriction of the smooth muscle and subsequent increase in airway resistance [57,58].

**Figure 2:**
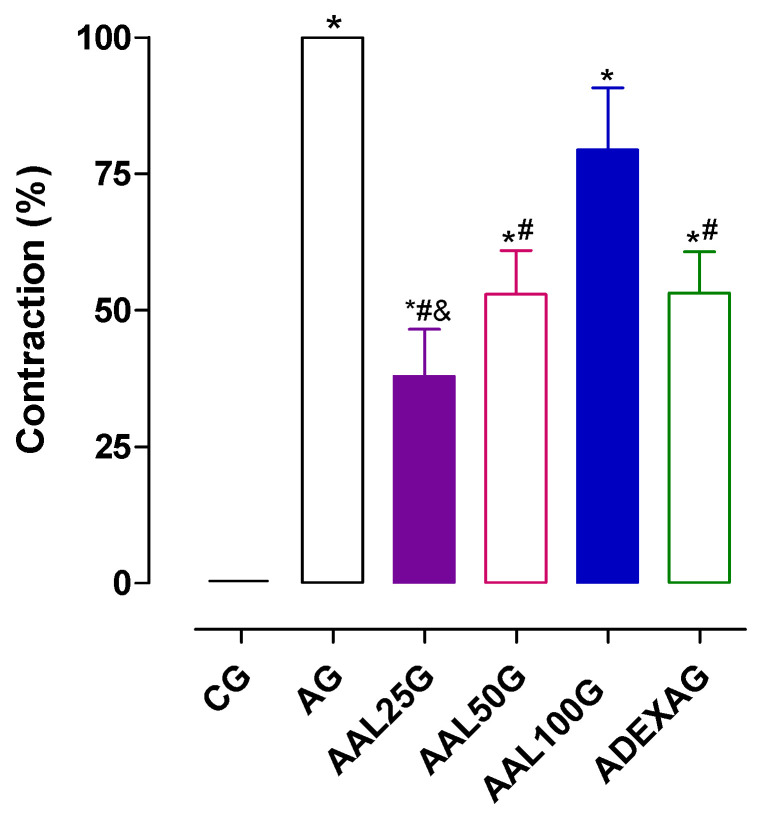
Contractile reactivity to OVA in the rat trachea of CG, AG, AAL25G, AAL50G, AAL100G, and ADEXAG. The symbols and vertical bars represent the mean and S.E.M., respectively. ANOVA one-way followed by Tukey’s post hoc test (n = 6). * *p* < 0.05 (CG vs. AG, AAL25G, AAL50G, AAL100G, and ADEXAG); ^#^ *p* < 0.05 (AG vs. AAL25G, AAL50G, AAL100G, and ADEXAG); ^&^ *p* < 0.05 (AAL100G vs. AAL25G).

Thus, the trachea of the sensitized and previously challenged rats with OVA were again exposed *in vitro* to 100 μg/mL of the same antigen. It was observed that prior sensitization and challenge with OVA in the AG promoted contractile reactivity in the trachea of these animals (E_max_ = 100%) compared to the CG, which showed no contractile reactivity to stimulation with 100 μg/mL of OVA (E_max_ = 0%), consistent with the expected results. This suggests that the reduction in ventilation minutes observed *in vivo* in the AG may be associated with a possible hyper-reactivity of the smooth muscle (Figure 2). Similar results were observed in the trachea of asthmatic rats after stimulation with OVA [49,59,60,61,62,63].

The increase in contractile reactivity observed in the AG (E_max_ = 100%) was prevented by treatment with lauric acid only at doses of 25 (E_max_ = 37.9 ± 8.7%) and 50 mg/kg (E_max_ = 53.0 ± 7.9%), showing no difference between the AAL100G (E_max_ = 79.4 ± 11.4%) and the AG (E_max_ = 100%).

These data suggest that lauric acid may partially inhibit the release of contractile factors by immune cells stimulated with OVA and smooth muscle contractility to reduce the bronchoconstriction observed in the GA (Figure 2). Similar results were observed for virgin coconut oil. This product contains lauric acid as a major component in a model of lung inflammation in guinea pigs, suggesting that lauric acid may be one of those responsible for this effect [6].

One of the most important classes for the treatment of asthma is corticosteroids, such as dexamethasone, fluticasone, or beclomethasone [64]. These drugs act on nuclear receptors, inhibiting the production of pro-inflammatory mediators and the enzyme phospholipase A_2_, resulting in the inhibition of cyclooxygenase-2, prostaglandins, and leukotrienes, as well as reducing the migration of neutrophils, eosinophils and mast cells to the sites affected by inflammation [65]. Based on this, it was observed that in the group treated with dexamethasone (ADEXAG) after re-exposure to OVA, the increase in contractile reactivity to this allergen was partially prevented (E_max_ = 53.2 ± 7.6%), suggesting a reduction in the release of these inflammatory mediators (Figure 2).

#### 2.5.3. Evaluation of Contractile Reactivity to KCl and CCh in Rat Trachea

The tracheal hyper-responsiveness to contractile stimuli, such as KCl, was evaluated. KCl is an electromechanical coupling agent that promotes contraction due to changes in membrane potential that lead to the accumulation of positive charges inside the cell and depolarization, which in turn stimulates the activation of voltage-dependent calcium channels (Ca_V_) and increases the cytosolic calcium concentration ([Ca^2^⁺]_c_) [66].

**Figure 3:**
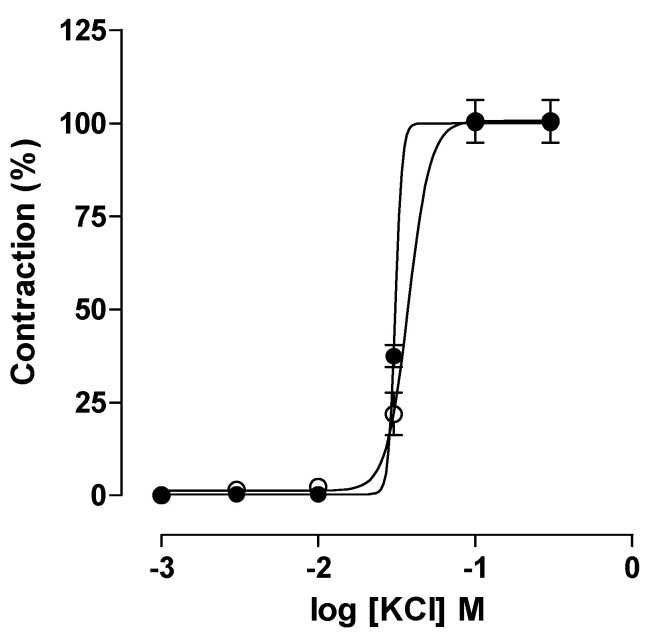
Cumulative concentration–response curves to potassium chloride (KCl) in the rat trachea of the CG (●) and AG (○) animals. The symbols and vertical bars represent the mean and S.E.M., respectively. Student’s *t*-test (n = 5).

It was observed that the trachea of the rat contracted in response to cumulative concentrations of KCl (10^−3^–3 × 10^−1^ M) without the alteration of efficacy or contractile potency in the AG animals (E_max_ = 99.8 ± 6.2% and EC_50_ = 3.5 ± 0.2 × 10^−2^ M; n = 5) compared to the CG (E_max_ = 100% and EC_50_ = 3.2 ± 0.03 × 10^−2^ M; n = 5) (Figure 3), suggesting that the electromechanical coupling of contraction does not contribute to the increased hyper-responsiveness of the airways observed in the induction of the asthma model.

Since there was no alteration in the contraction induced by electromechanical coupling in the trachea of AG rats, it was decided to investigate whether the pharmacomechanical coupling of contraction in this organ would be modified by performing cumulative concentration–response curves to carbachol (CCh), a muscarinic agonist and analog of acetylcholine (ACh), due to the cholinergic innervation being the primary factor responsible for maintaining airway tone [67].

Airway contraction is primarily controlled by the vagus nerve, which releases acetylcholine (ACh) and activates the post-junctional muscarinic ACh receptors in the smooth muscle of the airways, mainly M3. This neurotransmitter also contributes to the inflammation and remodeling of the airways and regulates the hypertrophy of smooth muscle cells in the airways [68]. Blocking these receptors is part of the clinical management of asthma through muscarinic antagonists, such as ipratropium or tiotropium, which function as bronchodilators [69].

Vasconcelos et al. (2019), in a model of chronic allergic pulmonary inflammation induced by OVA in guinea pigs [63], and Sousa et al. (2011) [70] and Ferreira et al. (2022) [49], in rats sensitized to this allergen, observed an increase only in the efficacy, not in the contractile potency to CCh of the trachea, in sensitized animals compared to a control group.

**Figure 4:**
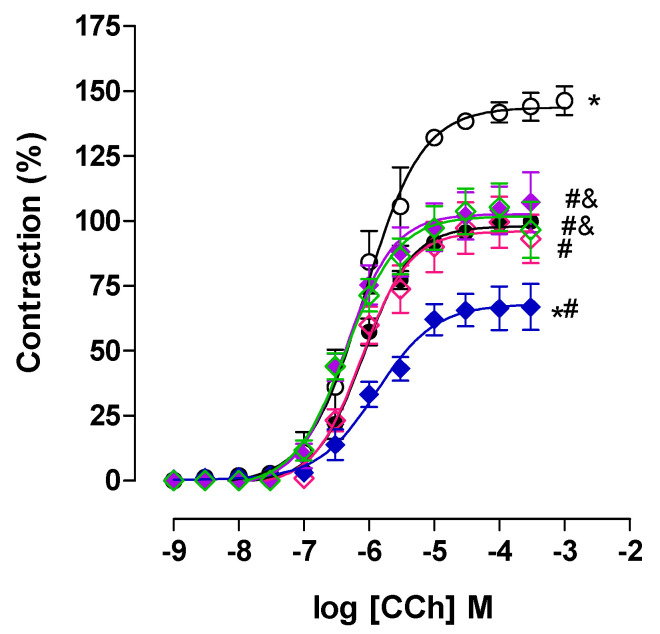
Cumulative concentration–response curves to carbachol (CCh) in the rat trachea of CG (●), AG (○), AAL25G (♦) [purple], AAL50G (◊) [pink], AAL100G (♦) [blue], and ADEXAG (◊) [green]. The symbols and vertical bars represent the mean and S.E.M., respectively. ANOVA one-way followed by Tukey’s post hoc test (n = 6). * *p* < 0.05 (CG vs. AG and AAL100G); ^#^ *p* < 0.05 (AG vs. AAL25G, AAL50G, AAL100G, and ADEXAG); ^&^ *p* < 0.05 (AAL100G vs. AAL25G and AGDEXA).

Similarly, in this study, it was observed that the trachea of the control group contracted in response to the addition of cumulative concentrations of CCh (10^−9^–3 × 10^−4^ M) (E_max_ = 100% and EC_50_ = 9.6 ± 1.7 × 10^−7^ M). In contrast, the AG animals exhibited an increase in contractile efficacy without alteration in potency (E_max_ = 146.3 ± 5.6% and EC_50_ = 1.3 ± 0.6 × 10^−6^ M) compared to the CG (Figure 4), indicating a greater contribution of the pharmacomechanical component to the hyper-responsiveness of the trachea in asthmatic rats.

Treatment with lauric acid at doses of 25 (E_max_ = 106.0 ± 9.0% and EC_50_ = 5.1 ± 0.4 × 10^−7^ M), 50 (E_max_ = 99.6 ± 9.7% and EC_50_ = 9.7 ± 2.1 × 10^−7^ M) and 100 mg/kg (E_max_ = 67.2 ± 6.9% and EC_50_ = 1.5 ± 0.6 × 10^−6^ M) prevented the increase in contractile efficacy observed in AG (E_max_ = 146.3 ± 5.6% and EC_50_ = 1.3 ± 0.6 × 10^−6^ M) without changing contractile power. Furthermore, AAL100G showed a reduction in contractile efficacy compared to the CG and other groups treated with lauric acid, indicating a more pronounced effect in reducing hyper-responsiveness at higher doses of this fatty acid (Figure 4). Thus, it is suggested that the preventive mechanism of the increase in hyper-responsiveness to lauric acid to CCh in the trachea of asthmatic rats appears to be through the negative modulation of the cholinergic pharmacomechanical pathway of contraction.

In animals treated with dexamethasone was observed prevention of hyper-responsiveness, similarly to what was observed in the groups treated with lauric acid, with a reduction in contractile efficacy and without change in potency (E_max_ = 105.3 ± 9.1% and EC_50_ = 5.3 ± 0.9 × 10^−7^ M), when compared with AG (E_max_ = 146.3 ± 5.6% and EC_50_ = 1.3 ± 0.6 × 10^−6^ M). Since LA is the major component of coconut oil, and it was observed by [6] that this product prevented the increase in hyper-responsiveness to CCh in the isolated trachea of guinea pigs sensitized with OVA in a similar manner to that observed in this study, and it is suggested that this effect of coconut oil may be mediated by lauric acid.

#### 2.5.4. Evaluation of Relaxant Reactivity to Nifedipine and Aminophylline in Rat Trachea

Considering that the contractile reactivity of the rat trachea to KCl was evaluated in the investigation of the participation of the electromechanical mechanism, whose concentration–response curves did not differ between the control and sensitized animals (Figure 3), it was decided to evaluate the involvement of the electromechanical coupling of relaxation using nifedipine, a Ca_V_ blocker [71].

**Figure 5:**
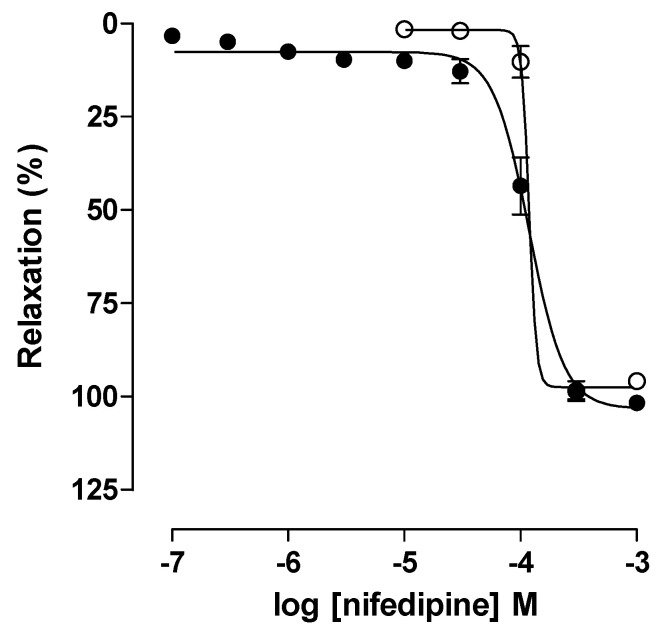
Cumulative concentration–response curves to nifedipine in pre-contracted rat trachea with 10^−5^ M CCh of the CG (●) and AG (○) animals. The symbols and vertical bars represent the mean and S.E.M., respectively. Student’s *t*-test (n = 5).

Nifedipine relaxed in a concentration-dependent manner (10^−7^–10^−3^ M) the rat trachea pre-contracted with 10^−5^ M CCh in the CG (E_max_ = 101.5 ± 0.8% and EC_50_ = 1.1 ± 0.2 × 10^−4^ M). No change in efficacy or relaxant potency was observed in the AG animals (E_max_ = 99.3 ± 2.4% and EC_50_ = 1.3 ± 0.1 × 10^−4^ M) when compared with CG (Figure 5).

Thus, it is suggested that this model of asthma induction by ovalbumin in Wistar rats does not alter the electromechanical component of relaxation, which is not the major pathway for altering tracheal hyper-responsiveness. These results are in agreement with what was observed for the contractile reactivity of the trachea to KCl in GA (Figure 3) and with those observed by Vasconcelos et al. (2019) in the trachea of guinea pigs with OVA-induced lung inflammation [63] and by Ferreira et al. (2022) [49], where no change in the relaxing reactivity of the trachea to nifedipine was observed.

In order to assess whether the disorders investigated in this study would alter the pharmacomechanical coupling of airway relaxation, aminophylline, a non-selective inhibitor of phosphodiesterases (PDEs) [72], was used. This type of coupling can occur through an increase in cyclic adenosine monophosphate (cAMP) and/or guanosine monophosphate (cGMP), which in turn activate PKA and PKG, respectively, leading to the relaxation of airway smooth muscle. PDEs are enzymes that inactivate, through hydrolysis, cAMP and cGMP into the respective 5′-monophosphates of adenosine (AMP) and guanosine (GMP). Thus, the inhibition of these enzymes favors the process of smooth muscle relaxation [73,74].

**Figure 6:**
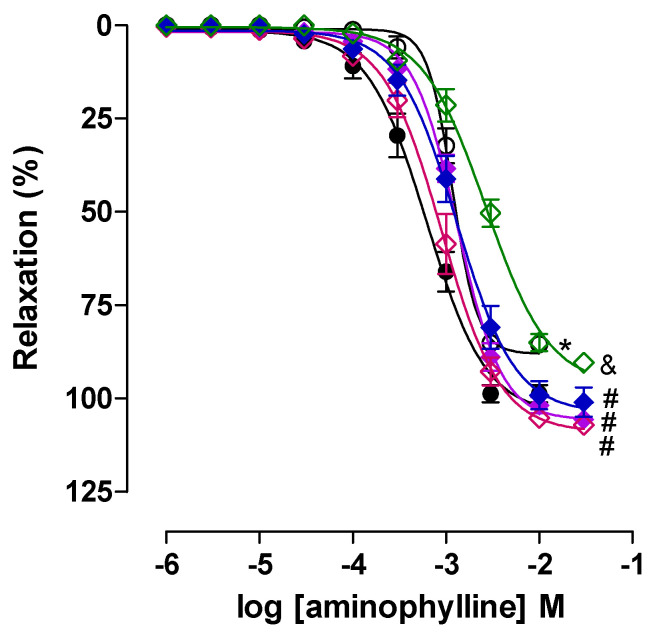
Cumulative concentration–response curves to aminophylline in pre-contracted rat trachea with 10^−5^ M CCh of CG (●), AG (○), AAL25G (♦) [purple], AAL50G (◊) [pink], AAL100G (♦) [blue], and ADEXAG (◊) [green]. The symbols and vertical bars represent the mean and S.E.M., respectively. ANOVA one-way followed by Tukey’s post hoc test (n = 6). * *p* > 0.05 (CG vs. AG); ^#^ *p* > 0.05 (AG vs. AAL25G, AAL50G, and AAL100G); ^&^ *p* > 0.05 (ADEXAG vs. AAL25G and AAL50G).

Aminophylline relaxed in a concentration-dependent manner (10^−6^–3 × 10^−2^ M) the rat trachea pre-contracted with 10^−5^ M CCh in the CG (E_max_ = 98.7 ± 2.3% and EC_50_ = 5.7 ± 0.7 × 10^−4^ M). In the AG animals, a reduction in the relaxing efficacy was observed without alteration in the potency (E_max_ = 85.1 ± 1.5% and EC_50_ = 1.0 ± 0.05 × 10^−3^ M) when compared with the CG (Figure 6).

Thus, a possible involvement of the pharmacomechanical mechanism of relaxation in the trachea of asthmatic rats is suggested, considering that relaxation was hampered in this group and that the reduction in bronchodilator mechanisms may be associated with the reduction in airflow observed in the *in vivo* protocol. Furthermore, it is reported that in the airway smooth muscle cells of asthmatic individuals, the expression of the PDE4 isoform is increased by up to two times when compared with that of non-asthmatic individuals [75], which justifies the increased degradation of cAMP and reduced relaxant activity in AG.

Different results were observed by Ferreira et al. (2022) [49], in which there was no significant difference in the relaxant efficacy of AG when compared with CG in a model of asthma induced by ovalbumin and exacerbated by obesity in Wistar rats.

Treatment with lauric acid at 25 (E_max_ = 105.6 ± 1.9% and EC_50_ = 1.3 ± 0.1 × 10^−3^ M), 50 (E_max_ = 101.4 ± 3.4% and EC_50_ = 8.2 ± 1.1 × 10^−4^ M), and 100 mg/kg (E_max_ = 100.6 ± 3.2% and EC_50_ = 2.4 ± 1.0 × 10^−3^ M) prevented the reduction in relaxant efficacy observed in AG (E_max_ = 85.1 ± 1.5% and EC_50_ = 1.0 ± 0.05 × 10^−3^ M), suggesting a possible negative modulation of the phosphodiesterase pathway in the preventive effect of lauric acid (Figure 6). In ADEXAG, there was no change in the efficacy or relaxing potency (Emax = 90.4 ± 0.9% and EC_50_ = 2.3 ± 0.2 × 10^−3^ M) compared to AG, showing a statistical difference in the relaxing efficacy compared to AAL25G and AAL50G, suggesting that treatment with dexamethasone does not alter the pharmacomechanical component of relaxation (Figure 6).

Other fatty acids have already shown potential for the treatment of asthma, such as oleic acid, a long-chain fatty acid that inhibits the infiltration of inflammatory cells; the production of interleukin (IL)-7, IL-5, and immunoglobulin E (IgE); and airway hyper-responsiveness [76].

### 2.6. Possible Pharmacological Targets of Lauric Acid In Silico

To evaluate the potential molecular targets of lauric acid involved in inflammation, remodeling, and airway smooth muscle reactivity, this compound was subjected to molecular docking simulations with 15 proteins. The results were generated using two scoring functions, the Moldock score and the Rerank score. More negative values indicated a higher likelihood of affinity for most scoring functions. The protein in which the compound achieved more negative binding energy values, or values close to the standard drug in at least one scoring function, was considered a potential target. Before performing the molecular docking simulations, the studied targets and their respective co-crystallized ligands were validated through redocking (Supplementary Material—Table S1).

Molecular docking techniques allow predicting and visualizing, with high precision, the interaction modes between molecules at the atomic level, providing detailed insights into mechanisms that are often challenging to observe by *in vitro* experimental methods. Furthermore, these tools can be used to improve functional macromolecules, making them more efficient, as well as to screen bioactive compounds in extensive databases containing millions of molecules [77,78].

By employing such strategies, the dependence on animal models for experimental validation is also significantly reduced, within the perspective of the 3Rs, with the use of these alternative computational models of substitution, promoting the optimization of the use of animals. This study will provide guidance for the possible mechanisms of action of AL that will be observed *in vitro* [79].

The redocking values for the proteins analyzed in this study can be found in Table S1, highlighting the RMSD (root mean square deviation) value, which is calculated using the coordinates of the heaviest atoms of the experimentally determined crystallographic structure and the docked pose, i.e., the quadratic mean distance between these atoms of the ligand in the crystal structure and the corresponding atoms in the docked pose.

Observing the RMSD value (best score) is a way to evaluate a method’s ability to find a ligand’s binding mode in a set of positions. For a docking simulation to be considered reliable, the RMSD value must be equal to or less than 2.0 Å. Therefore, an RMSD lower than 2.0 Å is widely accepted as a discriminator in reproducing a known binding mode, indicating whether the method was successful or not [80]. Accordingly, during the redocking analysis, it was observed that all the RMSD values were below 2.0 Å, meaning that the generated poses correctly positioned the ligand in the active site. This indicates that the program provided satisfactory values for docking validation (Supplementary Material—Table S1).

For proteins that do not have a co-crystallized ligand, the coordinates of the active site were determined through Web User-Friendly platforms, as well as by identifying the key residues essential for maintaining the activity of each target, as described in the reference article from the Protein Data Bank (PDB) platform. The platform used for this purpose was Bite Net-Skolteck I Molecule (https://sites.skoltech.ru/imolecule/tools/bitenet) (accessed on 23 July 2024). The enzymes in question included the voltage-dependent calcium channel (Ca_V_) (PDB: 3G43), which is represented by a unique crystalline structure of a 77-residue fragment from the carboxyl terminal of the α1 subunit (Ca_V_1.2), comprising a tandem of pre-IQ and IQ domains in complex with Ca^2+^-calmodulin (CaM) in two distinct binding modes. Important residues in the active site include glutamate (Glu) 104, methionine (Met) 109, and Met 124, which are the key residues involved in the modulation of Ca_V_1.2 by calmodulin [81]. Based on these residues, the coordinates used for calculation were X: 34.20; Y: 44.98, and Z: 39.49.

For the BKCa channel (PDB: 3NAF), it is notable that this corresponds to a structure with four intracellular subunits, each comprising two tandem RCK domains, assembled into a gating ring, with three observed Ca^2+^ binding sites. The residues involved in its inhibition include aspartic acid (Asp) 367, Glu 374, and Glu 399, which are located in the RCK domain [82]. The coordinates of the docking region were as follows: X: −55.58; Y: 42.44, and Z: 61.39.

The last target without a co-crystallized ligand was the enzyme 5-lipoxygenase (5-LOX) (PDB: 3O8Y), characterized as an iron-dependent metalloprotein with β-helix-rich N-terminal domains, a catalytic C-terminal domain, a substrate-binding pocket, a heme group, and a key residue for its inhibition, histidine (His) 367, which acts as a binding site for the direct inhibitors of the 5-LOX enzyme, such as zileuton [83]. The docking region coordinates were as follows: X: −12.24, Y: 25.37, and Z: −8.62.

Among the 15 proteins evaluated, those that presented greater interaction affinity compared to the positive control, based on the binding energy values, were the β_2_-adrenergic receptor, Ca_V_, BK_Ca_ and K_ATP_, adenylate cyclase, PKG, eNOS, iNOS, and COX-2, described below.

#### 2.6.1. Molecular Interaction Between Lauric Acid and the β_2_-Adrenergic Receptor (PDB: 2RH1)

β_2_-adrenergic receptors are widely expressed in the respiratory tract, particularly in the smooth muscle of the airways [84], and it has been reported that the desensitization and polymorphism of these receptors are involved in the severity of asthma [85,86]. Furthermore, they are highlighted as one of the main targets for asthma treatment [1]. Based on this, the molecular interaction of lauric acid with this receptor was evaluated.

The binding affinity values of the lauric acid–β_2_-adrenergic receptor complex (Supplementary Material—Table S2) were −98.8103 and −84.1847 kJ/mol, lower than those observed between the positive control, isoproterenol, a β-adrenergic agonist, and the β_2_-adrenergic receptor (−83.772 and −67.412 kJ/mol).

Regarding the active site of the β_2_-adrenergic receptor, lauric acid established two hydrogen bonds involving the asparagine (Asn) 312 and Asp 113 residues. Additionally, four hydrophobic interactions were identified with the amino acids valine (Val) 114, Val 117, phenylalanine (Phe) 290, and Phe 193, which are key interactions for maintaining the enzyme’s activity [87]. The positive control, isoproterenol, exhibited three hydrogen bonds with the serine (Ser) 204, Ser 203, and Asp 113 residues, as well as three hydrophobic interactions among the amino acids Phe 193, Phe 290, and Val 114 (Supplementary Material—Figure S6).

It was evident that there were common interactions between the two compounds under study, corresponding to the hydrogen bond with the residue Asp 113 and the hydrophobic interactions with the residues Val 114, Phe 290, and Phe 193 (Supplementary Material—Figure S6). Thus, it is suggested that β_2_-adrenergic receptors may be a potential activation target in the mechanism of action of lauric acid, contributing to airway relaxation.

#### 2.6.2. Molecular Interaction Between Lauric Acid and the Voltage-Dependent Calcium Channel (Ca_V_) (PDB: 3G43)

Given that airway hyper-responsiveness in asthma is associated with smooth muscle contraction, which in turn is regulated by cytosolic calcium concentration ([Ca^2+^]_c_), the importance of calcium homeostasis in asthma is emphasized [88,89]. Generally, the increase in [Ca^2+^]_c_ is regulated by the release of this ion from intracellular stores of the sarcoplasmic reticulum or through the influx of Ca^2+^ via a variety of ion channels in the plasma membrane, including the Ca_V_ channels [90]. Based on this, the molecular interaction of lauric acid with the Ca_V_ was evaluated.

The binding affinity values of the lauric acid–Ca_V_ macromolecule complex (Supplementary Material—Table S2) were −83.2615 and −60.699 kJ/mol, lower than those observed between the positive control, nifedipine, a Ca_V_ blocker, and the Ca_V_ (−73.8608 and −12.9409 kJ/mol). Lauric acid established only hydrophobic interactions with the residues arginine (Arg) 74, lysine (Lys) 75, methionine (Met) 71, Val 55, and alanine (Ala) 57. The positive control, nifedipine, established hydrogen bonds, hydrophobic interactions, and steric bonds (Supplementary Material—Figure S7).

The hydrophobic interactions corresponded to the residues Arg 74, Met 71, Val 55, and Ala 57 (establishing two interactions), in addition to a steric interaction through the residue Met 71 and hydrogen bonds through the residues Arg 74, glutamic acid (Glu) 67, and Val 55. Notably, similar hydrophobic interactions were observed between the two compounds under study, with the residues Arg 74, Met 71, Val 55, and Ala 57 (Supplementary Material—Figure S7). Thus, it is suggested that these channels may be potential targets for lauric acid through the negative modulation of Ca_V_, thereby contributing to the reduction in [Ca^2+^]_c_ and airway hyper-responsiveness.

#### 2.6.3. Molecular Interaction Between Lauric Acid and BK_Ca_ (PDB: 3NAF) and K_ATP_ (PDB: 6C3P)

Various subtypes of K^+^ channels can regulate the basal tone of smooth muscle, among which the most important identified in the airways are the BK_Ca_ and K_ATP_ channels. This importance has been established based on several studies that characterize K^+^ channel activators inducing hyperpolarization, reducing the infiltration of inflammatory cells, the levels of inflammatory cytokines, and hyper-responsiveness in animal models of allergic asthma [91]. Therefore, the molecular interaction of lauric acid with these channels was evaluated.

The binding affinity values of the lauric acid–BK_Ca_ and lauric acid–K_ATP_ macromolecule complexes were −112.247 and −93.4303 kJ/mol for BK_Ca_ and −92.7847 and −73.4965 kJ/mol for K_ATP_, respectively. These values were lower compared to those observed between the channels and their respective positive controls (Supplementary Material—Table S2) nicorandil, a BK_Ca_ blocker, and glibenclamide, a K_ATP_ blocker (−103.044 and −78.6007 kJ/mol; −130.484 and −22.5303 kJ/mol, respectively).

At the active site of BK_Ca_, lauric acid exhibited hydrogen bonding interactions with the amino acids Met 663, threonine (Thr) 946, and glycine (Gly) 944. The other interactions observed were with the residues Arg 664, isoleucine (Ile) 579, Arg 974, and cysteine (Cys) 975, with the last residue showing two interactions. The positive control, nicorandil, established hydrophobic interactions with the residues Arg 974 and Ile 581. Hydrogen bond interactions were established in greater numbers with the residues Asn 745 and Arg 974, leucine (Leu) 940, Cys 975, Arg 976, Leu 940, and Cys 975. Common interactions occurred between the two analyzed compounds, being hydrophobic through the residue Arg 974 (Supplementary Material—Figure S8).

The molecular docking simulation with the K_ATP_ channel demonstrated that lauric acid interacted through six binding sites, including three hydrogen bonding interactions with the amino acids Ser 1482 and Gln 774, which are important residues for stabilizing the SUR1 structure of the channel [92], and with Val 715 and Gly 716. Additionally, there were two hydrophobic interactions with the residues tryptophan (Trp) 688 and Met 409 (Supplementary Material—Figure S9).

The positive control glibenclamide exhibited a greater number of interactions, which were of hydrophobic, steric, and hydrogen bond types. The hydrogen bond interactions corresponded to the amino acids Trp 688, Ser 721, Ser 720, Gly 718, Gln 774, Gly 716, Ser 1482, and Asn 1480. The hydrophobic bonds were formed with Trp 688 and Met 409, while the steric interactions corresponded to Ser 1482 and Met 409. These residues are fundamental for stabilizing the SUR1 structure of the ATPase degeneracy site of the channel [92].

The residues Ser 1482, Gln 774, and Gly 716 (hydrogen bond interactions) and Trp 688 and Met 409 (hydrophobic interactions) were established by both compounds (Supplementary Material—Figure S9). Thus, it is suggested that the BK_Ca_ and K_ATP_ channels may be potential targets for activation in the mechanism of action of lauric acid.

#### 2.6.4. Molecular Interaction Between Lauric Acid and Adenylyl Cyclase (PDB: 4CLZ)

Based on the fact that the activation of adenylate cyclase leads to an increase in cAMP levels, an important second messenger directly related to the relaxation of smooth muscle in the airways [93], the molecular interaction of lauric acid with this enzyme was evaluated.

The binding affinity values of the ligand–macromolecule complex between lauric acid and adenylate cyclase (Supplementary Material—Table S2) were −67.0243 and −54.6923 kJ/mol, lower than those observed between the positive control, forskolin, an activator of adenylate cyclase, and adenylate cyclase (−40.7844 and −35.1598 kJ/mol).

Lauric acid formed three hydrophobic interactions with the amino acids Arg 15, Val 14, and Ala 18 (establishing two interactions), as well as two hydrogen bonds with the amino acids Ala 18 and Arg 250, and one steric interaction with the residue Val 14. The positive control forskolin formed four hydrophobic interactions with the residues Phe 8, Val 14, and Ala 18, and also established two hydrogen bonds with the amino acids Asp 10 (establishing two interactions) and Arg 15 (establishing three interactions) (Supplementary Material—Figure S10).

It is noteworthy that coinciding interactions occurred between the molecules under study, involving the hydrophobic interaction encompassing the residue Ala 18. Additionally, lauric acid formed a hydrophobic interaction with the amino acid Val 14, which was also presented by forskolin (Supplementary Material—Figure S10). Thus, it is suggested that adenylate cyclase may be a potential target for activation in the mechanism of action of lauric acid, contributing to the relaxation of the smooth muscle in the airways.

#### 2.6.5. Molecular Interaction Between Lauric Acid and cGMP-Dependent Protein Kinase (PKG) (PDB: 6BQ8)

Knowing that the activation of soluble guanylyl cyclase (sGC) leads to an increase in cGMP levels, which in turn activates PKG, and that this enzyme phosphorylates various targets that result in the relaxation of smooth muscle in the airways [90], the molecular interaction of lauric acid with this enzyme was evaluated.

The binding affinity values of the ligand–macromolecule complex between lauric acid and PKG (Supplementary Material—Table S2) were −104.44 and −85.5268 kJ/mol, showing a lower value only in the Rerank score function compared to those observed between the positive control, cGMP, and the enzyme PKG (−120.89 and −37.4022 kJ/mol).

In the mechanism of PKG, lauric acid established three hydrogen bonds with Lys 358, Leu 408, and Gln 335, in addition to five hydrophobic interactions with Lys 358, two interactions with Ala 368, Arg 366, and Leu 408, the last two being key residues for the inhibition of the enzyme [94], and Ala 359 (Supplementary Material—Figure S11).

The ligand cGMP established hydrogen, steric, and hydrophobic interactions. The hydrogen interactions involved the residues Ala 368, Ala 359, Ser 367, Arg 366, Gly 356, Lys 358, and Asp 412. The hydrophobic interactions corresponded to the amino acids Ala 368, Leu 408, and Val 333. The steric interactions occurred with Gly 356 and Lys 358. The interactions with Lys 358 (hydrogen bond), Leu 408, and Ala 368 (steric interactions) were present in both compounds (Supplementary Material—Figure S11). Thus, it is suggested that this enzyme may also be a potential target for activation in the mechanism of action of lauric acid.

#### 2.6.6. Molecular Interaction Between Lauric Acid and Endothelial Nitric Oxide Synthase (eNOS) (PDB: 1M9J) and Inducible Nitric Oxide Synthase (iNOS) (PDB: 4NOS)

In the respiratory tract, there are three isoforms of nitric oxide synthase (NOS), with the endothelial isoform (eNOS) located in the epithelium of the bronchi, alveoli, and trachea. The inducible isoform (iNOS) is found in macrophages, neutrophils, fibroblasts, and vascular and epithelial endothelial cells of the airways, and the neuronal isoform (nNOS) located in nerves that innervate the lamina propria, blood vessels, and smooth muscle of the airways in both humans and animals. Nitric oxide (NO) is, therefore, an important mediator of bronchodilation in the airways [95,96]. Thus, the molecular interaction of lauric acid with these isoforms of NOS was evaluated.

The binding affinity values of the ligand–macromolecule complex between lauric acid and eNOS (−95.2229 and −78.9643 kJ/mol) and iNOS (−98.2345 and −83.3349 kJ/mol) were lower than those observed between the positive controls, GW-274150 (an eNOS inhibitor) and 1400 W (an iNOS enzyme inhibitor), and their respective isoforms, eNOS (−79.1829 and −64.7938 kJ/mol) and iNOS (−87.3824 and −74.6874 kJ/mol) (Supplementary Material—Table S2).

Lauric acid established hydrogen bonds and steric interactions with the active site of the endothelial isoform (eNOS), with predominant hydrophobic interactions involving the amino acids proline (Pro) 334, Trp 178 (2 interactions), Leu 193, and Phe 353. The positive control GW-274150 formed hydrogen bonds through the residue Trp 356, and hydrophobic interactions were observed through the residue Pro 334, along with a steric interaction involving the residue Phe 353. In this mechanism, similar interactions were noted between the studied molecules corresponding to the residue Pro 334 in the hydrophobic interaction (Supplementary Material—Figure S12).

Regarding the inducible isoform (iNOS), lauric acid formed hydrogen bonds and hydrophobic interactions with the active site of the iNOS enzyme. The residue Trp 463 was involved in the established hydrogen bond, while the amino acids His 477, Ile 462, Met 120, Trp 461, Phe 476, and Trp 463 were associated with hydrophobic interactions, with the residue Trp 463 being important for the formation of the enzyme’s catalytic dimer [97].

The positive control 1400 W established two steric interactions through the residues Arg 381 and Met 120, as well as three hydrogen bonds with the residues Trp 463, Ile 462, and Phe 476, and four hydrophobic interactions through the residues Phe 476, Met 120, Ile 462, and Trp 463. Similarly to the results demonstrated in the eNOS enzyme, a coincidence in the hydrogen bond formed by the residue Trp 463 was observed, along with similar hydrophobic interactions involving the residues Trp 463, Phe 476, and Met 120 (Supplementary Material—Figure S13). Thus, it is suggested that the NOS isoforms may be potential activation targets in the mechanism of action of lauric acid.

#### 2.6.7. Molecular Interaction Between Lauric Acid and Cyclooxygenase-2 (PDB: 5IKR)

Cyclooxygenase-2 (COX-2) is responsible for metabolizing arachidonic acid to form lipid mediators known as prostanoids. This enzyme is expressed in epithelial cells and macrophages and is inducible by cytokines and other inflammatory mediators, promoting the synthesis of inflammatory prostaglandins during asthma [98].

There is an increased production of prostaglandins, such as PGD_2_ and PGE_2_, during the allergic inflammation of the airways, and they are known to participate in the pathogenesis of asthma in humans [99,100] and in animal models [101,102]. Given the importance of these prostanoids in the contraction of airway smooth muscle [103], the molecular interaction of lauric acid with this enzyme was evaluated.

The binding affinity values of the ligand–macromolecule complex between lauric acid and COX-2 (Supplementary Material—Table S2) were −97.5696 and −81.663 kJ/mol, which are lower than those observed with the positive control, etoricoxib (a COX-2 inhibitor), and the COX-2 enzyme (−90.7927 and −82.8298 kJ/mol).

It is important to mention that in the study conducted by Jack, Asruddin, and Bhawani (2022) regarding the target COX-2, when compared to the positive controls acetylsalicylic acid, ibuprofen, and paracetamol, lauric acid did not exhibit lower binding energy scores than these compounds, corresponding to −6.62 kcal/mol. In contrast, the control drugs displayed more negative binding energy values of −7.14, −7.72, and −6.76 kcal/mol, respectively. However, regarding etoricoxib, the positive control of this study, it showed greater affinity according to the mentioned binding energy scores [104].

Furthermore, the simulation analysis demonstrated that lauric acid formed two hydrogen bonds involving the residues Ser 530 and tyrosine (Tyr) 385, which are key interactions for inhibiting the enzyme [105]. Additionally, it established seven hydrophobic interactions with the amino acids Trp 387, Tyr 385, Ala 527, Leu 352, Val 523, Val 349, and Tyr 355 (Supplementary Material—Figure S14).

Regarding etoricoxib, steric and hydrophobic interactions were established with the active site of the protein under study. Steric interactions involved the residues Leu 392, Val 523, and Phe 518, while hydrophobic interactions were more numerous, being Leu 392, Val 523, Val 116, Leu 359, Leu 531, Ala 527, Val 349, Met 522, Leu 384, Trp 387 and Tyr 385.

It is worth noting that, unlike etoricoxib, lauric acid established hydrogen bonds, which is very important since this type of molecular interaction is considered the strongest. Furthermore, it was found that similar bonds occurred comprising hydrophobic interactions through residues Trp 387, Tyr 385, Ala 527, Val 523, and Val 349 (Supplementary Material—Figure S14). Thus, it is suggested that COX-2 is a possible target of inhibition in the mechanism of action of lauric acid, inhibiting the formation of contractile prostanoids.

Molecular docking studies, despite their effectiveness in predicting interactions between drugs and biological targets, do not fully simulate the real biological environment. One of the main limitations of this method is the ability to reliably simulate the pharmacokinetic aspects and pharmacogenetic specifications that significantly affect the interaction between the ligand and the target. In addition, many docking models consider proteins as rigid structures, while in reality, proteins can undergo substantial conformational changes during interaction with the ligand [106].

Based on the results obtained, these molecular docking studies point to future *in vitro* results that will be investigated regarding the mechanism of action of LA. Thus, the integration of *in vitro* and *in vivo* studies as a validation step of *in silico* methods is an indispensable part of the drug discovery process.

## 3. Materials and Methods

### 3.1. Animals

Wistar rats (*Rattus norvegicus*) weighing between 250 and 300 g, sourced from the Animal Production Unit (UPA) of the Instituto de Pesquisa em Fármacos e Medicamentos (IPeFarM) at the Universidade Federal da Paraíba (UFPB), were used. The animals were kept under controlled temperature conditions (22 ± 1 °C) and a 12 h light–dark cycle with free access to food and water. Experimental procedures were conducted in accordance with the principles of the guidelines for the ethical use of animals in applied etiology studies [107] and the Brazilian Guide for the Production, Maintenance, or Use of Animals in Educational or Scientific Research Activities by the National Council for the Control of Animal Experimentation (CONCEA) [108]. The experimental procedures were approved by the Animal Use Ethics Committee (CEUA) of UFPB (n° 9310040522).

### 3.2. Chemicals

Sodium chloride (NaCl), potassium chloride (KCl), magnesium sulfate (MgSO_4_), potassium phosphate (KH_2_PO_4_), calcium chloride (CaCl_2_), glucose, sodium bicarbonate (NaHCO_3_), hydrochloric acid (HCl), and sodium hydroxide (NaOH) were obtained from Êxodo Científica (Sumaré, Brazil).

Lauric acid, aluminum hydroxide (Al(OH)_3_), ovalbumin (OVA) (grade II and V), carbamylcholine hydrochloride (CCh), nifedipine, and aminophylline were obtained from Sigma-Aldrich (Sao Paulo, Brazil). Tween^®^ 80 was obtained from Fischer BioReagents. Dexamethasone was purchased from Aché Laboratórios Farmacêuticos (Guarulhos, São Paulo, Brazil). Ketamine and xylazine were purchased from Syntec (Barueri, São Paulo, Brazil). Biochemical analyses were performed using specific reagent kits from Labtest (Lagoa Santa, Brazil).

### 3.3. Physiological Solutions

For the tracheal reactivity protocols, a Krebs nutrient solution was used in an organ bath, adjusted to pH 7.4 (with a solution of HCl or NaOH, 1 N), aerated with a carbogen mixture (95% O_2_ and 5% CO_2_) and maintained at 37 °C. It had the following composition in mM: NaCl (118.0), KCl (4.5), MgSO_4_ (5.7), KH_2_PO_4_ (1.1), CaCl_2_ (2.5), glucose (11.0), and NaHCO_3_ (25.0).

### 3.4. Experimental Groups

For the repeated dose toxicity protocol, the animals were randomly divided into 4 groups, with 5 animals each: Groups 1 and 2 = control group of females and males (CG): treated with NaCl 0.9% + Tween^®^ 80; Groups 3 and 4 = lauric acid group 100 mg/kg of females and males (AL100G): treated with lauric acid at a dose of 100 mg/kg.

For the protocols involving asthmatic animals, they were randomly divided into 5 experimental groups, with 5 male rats each: control group (CG) = not sensitized and treated with NaCl 0.9% + Tween^®^ 80; asthmatic group (AG) = sensitized with OVA and treated with NaCl 0.9% + Tween^®^ 80; asthmatic lauric acid 25 mg/kg group (AAL25G) = sensitized with OVA and treated with 25 mg/kg of lauric acid; asthmatic lauric acid 50 mg/kg group (AAL50G) = sensitized with OVA and treated with 50 mg/kg of lauric acid; asthmatic lauric acid 100 mg/kg group (AAL100G) = sensitized with OVA and treated with 100 mg/kg of lauric acid; and asthmatic dexamethasone 5 mg/kg group (ADEXAG) = sensitized with OVA and treated with 5 mg/kg of dexamethasone.

The doses of lauric acid were based on previous studies [109].

### 3.5. Preparation of Lauric Acid

Tween^®^ 80 was used as a solubilizing agent, and the solution was placed at 40 °C in a water bath and kept under constant stirring until the substance was completely solubilized. Subsequently, a saline solution was added for the administration of lauric acid.

### 3.6. Assessment of Repeated-Dose Toxicity and Behavioral Pharmacological Screening of Lauric Acid

To assess the oral toxicity of repeated doses, the methodology described in guideline n° 407 of the Organisation for Economic Co-operation and Development [110] was used. In this study, 20 animals were utilized, distributed into 2 groups, each with 10 animals, consisting of 5 males and 5 nulliparous, non-pregnant females. The animals were treated daily by oral gavage with lauric acid at a dose of 100 mg/kg (AL100G) or with saline and Tween^®^ 80 (CG) for 28 days. After the treatment period and a 28-day observation, the animals were monitored for an additional 14 days without treatment to detect late occurrence or persistence and recovery from potential toxic effects.

Furthermore, behavioral parameters assessing central nervous system (CNS) stimulation (such as increased aggression and ambulation), CNS depression (such as analgesia and anesthesia), and activity on the peripheral nervous system (such as cyanosis and piloerection) were evaluated before the onset of administrations and for up to 4 h, followed by periodic evaluations until the end of the protocol [27] (Supplementary Material—Table S3).

#### 3.6.1. Assessment of Weight, Food and Water Intake, and Organ Weight

The animals were weighed on a semi-analytical balance model BG4001, from Gehaka (São Paulo, Brazil), before treatments and then weekly, with the average weight gain calculated for the animals. To quantify food and water intake, consumption was calculated by the difference between the amount offered and the remaining amount that was not ingested. After euthanasia with ketamine (180 mg/kg) and xylazine (30 mg/kg) via intraperitoneal injection, the heart, kidneys, liver, spleen, stomach, lungs, uterus, ovaries, testes, and intestines were removed, weighed, and evaluated macroscopically.

#### 3.6.2. Hematological and Biochemical Analysis

After the last day of observation, on the 42nd day, the animals were euthanized and after that, blood was collected by the venipuncture of the inferior abdominal vena cava using heparinized syringes.

In the hematological analysis, blood was collected into tubes containing ethylenediaminetetraacetic acid (EDTA) for the erythrogram, leukogram, and platelet count. The SCIL Vet abc-Bomba ABX^®^ automatic veterinary cell was used. The erythrogram included the counting of red blood cells, and determination of hemoglobin, hematocrit, mean corpuscular volume (MCV), mean corpuscular hemoglobin (MCH), and mean corpuscular hemoglobin concentration (MCHC). The leukogram included the total and differential leukocyte counts.

For the evaluation of biochemical parameters, the blood was collected into Microtainer Becton Dickson^®^ tubes (Becton, Dickinson and Company (BD), Franklin Lakes, NJ, USA) with a separator gel, which was centrifuged for 10 min at 3500 rpm to obtain serum. The ChemWell-T^®^ automated analyzer was used to quantify glucose, total cholesterol, aspartate aminotransferase (AST), alanine aminotransferase (ALT), uric acid, and urea.

### 3.7. Evaluation of the Effect of Lauric Acid on Airway Hyper-Responsiveness

#### 3.7.1. Sensitization and Challenges with Ovalbumin for Asthma Induction

**Figure 7:**
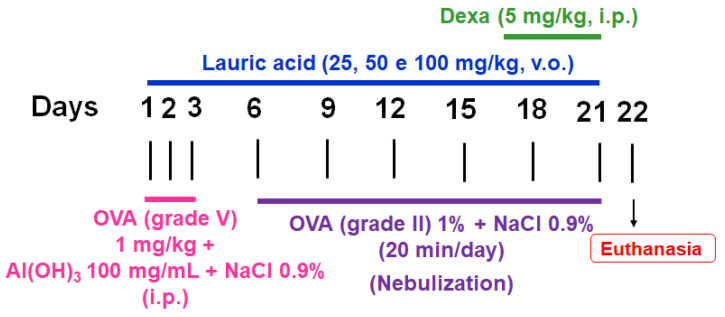
Flowchart and timeline of the study design for asthma induction on rats. OVA: ovalbumin; Al(OH)_3_: aluminum hydroxide; Dexa: dexamethasone; i.p.: intraperitoneally; v.o.: oral route.

For the sensitization protocol, on days 1–3 of the experiment, the animals received intraperitoneal (i.p.) injections of 1 mg/kg/day of ovalbumin (OVA) (grade V) solubilized in sterile NaCl 0.9% using 100 mg/mL of aluminum hydroxide (Al(OH)_3_) as an adjuvant. On days 6, 9, 12, 15, 18, and 21, the animals were individually placed in a closed polyacrylic chamber connected to an ultrasonic nebulizer. They were then challenged with 1% OVA (grade II) for up to 20 min daily. The non-sensitized animals underwent the same process but were administered only sterile NaCl 0.9% for both the i.p. injections and nebulizations. All the animals were euthanized 24 h after the last challenge with OVA or NaCl 0.9% (day 22). Throughout the asthma induction, the asthmatic group animals received daily doses of lauric acid via oral gavage (v.o.). The animals in the ADEXAG during the last 5 days of disease induction received 5 mg/kg/day of dexamethasone i.p., while the other groups received NaCl 0,9% via the same route [111,112] (Figure 7).

#### 3.7.2. Respiratory Parameters Analysis

On days 1, 12, and 21 of the asthma induction protocol, the animals were subjected to the measurements of the respiratory rate, tidal volume, and minute volume using whole-body plethysmography, adapted from the literature [113,114].

#### 3.7.3. Assessment of Tracheal Contractile and Relaxant Reactivity

##### Contractile Reactivity to Ovalbumin in Rat Trachea: Schultz–Dale Reaction

The rats were euthanized as previously described. The trachea was divided into rings, and to record isometric contractions, the organs were suspended in isolated organ bath chambers (6 mL), model BOI-04, and connected to isometric force transducers, model TIM 05, coupled to an amplifier, model AECAD04F. This, in turn, was connected to a digital acquisition system with the AQCAD software version 2.5.0 for data acquisition and ANCAD for analysis. The system contained a BT 60 thermostatic pump that controlled the bath temperature. All the devices were purchased from AVS Projetos (São Paulo, Brazil).

The trachea was stimulated with 100 µg/mL OVA and the amplitude of contraction was compared between CG, AG, AALG, and ADEXAG [115,116].

##### Contractile Reactivity to KCl and CCh in Rat Trachea

Two cumulative concentration–response curves to KCl, an electromechanical agent [117], or to CCh, a pharmacomechanical agent [67], were induced. Contractile reactivity was calculated based on the mean amplitude of the response of the rat trachea of the CG, and comparisons were made between the groups based on the values of maximum effect (E_max_) and the concentration of a substance that produces 50% of its E_max_ (EC_50_) of the contractile agents, calculated by nonlinear regression.

##### Relaxant Reactivity to Nifedipine and Aminophylline in Rat Trachea

A contraction with 10^−5^ M CCh was induced and over the tonic component, nifedipine, a voltage-dependent calcium channel blocker [71], or aminophylline, a phosphodiesterase inhibitor [72], was added in cumulative concentrations until their E_max_ was reached.

The relaxant response was expressed as the percentage reversed from the initial contraction produced by CCh. Comparisons were made between the groups based on the E_max_ and EC_50_ values of the relaxant agents, calculated from the cumulative concentration–response curves obtained by nonlinear regression.

### 3.8. Molecular Docking

The 3D structures of the enzymes were obtained from the Protein Data Bank (PDB) (https://www.rcsb.org/) (accessed on 10 January 2024) [118]. The analyses were carried out in January 2022 and updated in July 2024. Among the analyzed proteins (Supplementary Material—Table S3), those selected for interaction studies with lauric acid were the β_2_-adrenergic receptor, the voltage-dependent calcium channel (Ca_V_), the large conductance calcium-activated potassium channels (BK_Ca_) and ATP-sensitive potassium channels (K_ATP_), adenylate cyclase (AC), cGMP-dependent protein kinase (PKG), endothelial nitric oxide synthase (eNOS) and inducible nitric oxide synthase (iNOS), and cyclooxygenase-2 (COX-2).

All the water molecules were removed from the crystalline structure of the selected proteins, and the root mean square deviation (RMSD) was calculated based on the poses, indicating the degree of reliability of the fit. The RMSD provides the proximity of the connection to the experimental structure and is considered successful if the value is below 2.0 Å.

Molecular docking simulations were performed using the Molegro Virtual Docker v.6.0.1 (MVD) software with predefined parameters [119]. The ligand complexed with the enzyme was used to define the active site. Next, the structure of lauric acid was imported to analyze the system’s stability through the interactions identified with the enzyme’s active site using the Moldock and Rerank score energy value as a reference.

For this purpose, the Moldock SE (Simplex Evolution) algorithm was employed with the following parameters: a total of 10 runs with a maximum of 1500 interactions using a population of 50 individuals, 2000 minimization steps for each flexible residue, and 2000 global minimization steps per run. The MolDock score (GRID) scoring function was used to calculate the docking energy values. A GRID was set at 0.3 Å, and the search sphere was fixed at a radius of 15 Å. For the ligand energy analysis, internal electrostatic interactions, internal hydrogen bonds, and sp^2^-sp^2^ torsions were evaluated [120].

### 3.9. Statistical Analysis

The results were expressed as the percentage of the mean and the standard error of the mean (S.E.M.), and the E_max_ and EC_50_ were compared and analyzed statistically using Student’s *t*-test or one-way analysis of variance (ANOVA) followed by the Tukey post hoc test. The null hypothesis was rejected when *p* < 0.05. All the data were analyzed using GraphPad Prism^®^ version 5.01.

## 4. Conclusions

Based on the results obtained, it is concluded that lauric acid administered at a dose of 100 mg/kg in repeated doses presents low toxicity considering the absence of mortality and changes related to food and water consumption, organ weight, and significant hematological and biochemical parameters, ensuring the safety of this drug for *in vivo* use in other experimental protocols.

In addition, lauric acid prevented tracheal contractile and relaxant hyper-responsiveness in Wistar rats in an ovalbumin-induced allergic asthma model. In *in silico* studies, it was shown that lauric acid has several possible molecular targets of interaction, among them, the β_2_-adrenergic receptor, Ca_V_, BK_Ca_ and K_ATP_, adenylate cyclase, PKG, eNOS, iNOS, and COX-2. These targets of interaction involved in smooth muscle reactivity and airway inflammation should be confirmed in future *in vitro* experimental protocols.

## Figures and Tables

**Table 1:** Weight evolution (g) of female and male Wistar rats from the control group (CG) and the group subjected to repeated-dose treatment with lauric acid at a dose of 100 mg/kg/day (AL100G).

Weight Evolution	CG (g)		AL100G (g)	
	Female	Male	Female	Male
Weight initial	210.4 ± 5.5	343.2 ± 10.1	216.8 ± 6.1	345.0 ± 10.6
Weight final	258.4 ± 7.5 *	457.0 ± 15.7 *	263.8 ± 5.1 *	456.6 ± 6.9 *
Weight gain	48.0 ± 4.2	113.8 ± 8.2	47.0 ± 2.9	111.6 ± 9.2

Data are expressed as the mean ± S.E.M. Student’s *t*-test, * *p* < 0.05 (Weight initial vs. final) (n = 5).

**Table 2:** The average weekly consumption of water and feed of female and male Wistar rats in the control group (CG) and the group subjected to repeated dose treatment with lauric acid at 100 mg/kg/day (AL100G).

Consumption	CG		AL100G	
	Female	Male	Female	Male
Food (g)	178.2 ± 24.8	254.8 ± 16.9	162.9 ± 22.3	236.6 ± 20.3
Water (mL)	103.2 ± 5.6	180.3 ± 12.5	90.2 ± 5.5	145.6 ± 10.4

Data are expressed as the mean ± S.E.M. Student’s *t*-test (n = 5).

**Table 3:** The relative weight of the internal organs of female and male Wistar rats in the control group (CG) and the group subjected to repeated dose treatment with lauric acid at 100 mg/kg/day (AL100G).

Organs	CG (g/100 g)		AL100G (g/100 g)	
	Female	Male	Female	Male
Heart	0.33 ± 0.01	0.28 ± 0.01	0.35 ± 0.01	0.29 ± 0.01
Kidneys	0.80 ± 0.03	0.77 ± 0.04	0.82 ± 0.03	0.87 ± 0.04
Liver	3.52 ± 0.15	3.39 ± 0.04	3.53 ± 0.12	3.54 ± 0.14
Spleen	0.28 ± 0.01	0.25 ± 0.01	0.28 ± 0.01	0.25 ± 0.01
Stomach	0.95 ± 0.11	0.68 ± 0.05	0.76 ± 0.06	0.71 ± 0.08
Lungs	0.64 ± 0.04	0.50 ± 0.04	0.68 ± 0.05	0.63 ± 0.05
Uterus and ovaries	1.22 ± 0.13	-	1.39 ± 0.13	-
Testicles	-	1.19 ± 0.07	-	1.19 ± 0.15
Intestines	6.03 ± 0.36	5.49 ± 0.51	6.03 ± 0.36	5.52 ± 0.24

Data are expressed as the mean ± S.E.M. Student’s *t*-test (n = 5).

**Table 4:** Hematological parameters were obtained from the blood of male Wistar rats from the control group (CG) and the group subjected to repeated dose treatment with lauric acid at 100 mg/kg/day (AL100G).

Parameters	CG	AL100G	Reference Values *
Red blood cells (10^6^/μL)	7.9 ± 0.2	8.3 ± 0.4	6.0–10.0
Hematocrit (g/dL)	41.6 ± 0.8	43.4 ± 2.2	39.0–55.0
Hemoglobin (%)	14.1 ± 0.3	14.9 ± 0.7	11.0–19.5
M.C.V. (fL)	52.7 ± 0.8	52.0 ± 0.4	55.0–65.0
M.C.H. (%)	17.8 ± 0.2	17.8 ± 0.2	18.3–19.5
C.H.C.M. (pg)	33.9 ± 0.09	34.2 ± 0.2	28.2–35.4
Leukocyts (10^3^/mm^3^)	8.0 ± 0.7	8.7 ± 0.9	6.0–15.0
Myelocytes (%)	0	0	0
Metamyelocytes (%)	0	0	0
Rods (%)	0	0	0
Segmented (%)	29.4 ± 1.5	30.4 ± 1.2	9.0–34.0
Eosinophils (%)	0	0	0–6.0
Basophils (%)	0	0	0–1.5
Lymphocytes (%)	68.6 ± 1.5	67.4 ± 1.0	65.0–85.0
Monocytes (%)	2.0	2.2 ± 0.2	0–5.0
Platelets (10^3^/mm^3^)	1069.0 ± 65.6	1107.0 ± 77.7	500.0–1300.0

Data are expressed as the mean ± S.E.M. Student’s *t*-test (n = 5). M.C.V. = mean corpuscular volume; M.C.H. = mean corpuscular hemoglobin; C.H.C.M. = mean corpuscular hemoglobin concentration. * [33].

**Table 5:** Hematological parameters obtained from the blood of female Wistar rats from the control group (CG) and the group subjected to repeated dose treatment with lauric acid at a dose of 100 mg/kg/day (AL100G).

Parameters	CG	AL100G	Reference Values *
Red blood cells (10^6^/μL)	7.3 ± 0.2	7.3 ± 0.2	6.0–10.0
Hematocrit (g/dL)	37.6 ± 1.4	38.8 ± 0.8	39.0–55.0
Hemoglobin (%)	12.9 ± 0.5	13.5 ± 0.3	11.0–19.5
M.C.V. (fL)	51.3 ± 0.8	53.6 ± 0.7	55.0–65.0
M.C.H. (%)	17.5 ± 0.3	18.6 ± 0.1 *	18.3–19.5
C.H.C.M. (pg)	34.2 ± 0.1	34.9 ± 0.3	28.2–35.4
Leukocyts (10^3^/mm^3^)	5.7 ± 0.8	3.8 ± 0.5	6.0–15.0
Myelocytes (%)	0	0	0
Metamyelocytes (%)	0	0	0
Rods (%)	0	0	0
Segmented (%)	23.0 ± 1.7	27.8 ± 0.6 *	9.0–34.0
Eosinophils (%)	0	0	0–6.0
Basophils (%)	0	0	0–1.5
Lymphocytes (%)	74.6 ± 1.9	69.8 ± 0.6 *	65.0–85.0
Monocytes (%)	2.4 ± 0.2	2.4 ± 0.2	0–5.0
Platelets (10^3^/mm^3^)	765.0 ± 49.0	1075.0 ± 53.9 *	500.0–1300.0

Data are expressed as the mean ± S.E.M. Student’s *t*-test, * *p* < 0.05 (n = 5). M.C.V. = mean corpuscular volume; M.C.H. = mean corpuscular hemoglobin; C.H.C.M. = mean corpuscular hemoglobin concentration.

**Table 6:** Biochemical parameters obtained from the blood of male Wistar rats from the control group (CG) and the group subjected to repeated dose treatment with lauric acid at a dose of 100 mg/kg/day (AL100G).

Parameters	CG	AL100G	Reference Values *
Glucose (mg/dL)	157.6 ± 15.5	167.0 ± 16.7	85.0–130.0
Cholesterol (mg/dL)	66.4 ± 2.3	85.4 ± 5.3 *	46.0–92.0
ALT/SGPT (U/L)	36.0 ± 2.7	43.2 ± 6.7	17.0–50.0
AST/SGOT (U/L)	82.0 ± 6.6	100.6 ± 12.8	39.0–92.0
Uric acid (U/L)	2.6 ± 0.8	3.5 ± 0.4	0.66–1.60
Urea (U/L)	46.8 ± 1.4	50.8 ± 3.1	32.0–54.0

Data are expressed as the mean ± S.E.M. Student’s *t*-test, * *p* < 0.05 (n = 5). * [33].

**Table 7:** Biochemical parameters obtained from the blood of female Wistar rats from the control group (CG) and the group subjected to repeated dose treatment with lauric acid at a dose of 100 mg/kg/day (AL100G).

Parameters	CG	AL100G	Reference Values *
Glucose (mg/dL)	150.6 ± 23.9	133.8 ± 30.4	85.0–130.0
Cholesterol (mg/dL)	93.0 ± 6.8	75.4 ± 8.9	46.0–92.0
ALT/SGPT (U/L)	36.0 ± 1.6	50.4 ± 9.9	17.0–50.0
AST/SGOT (U/L)	63.8 ± 17.2	59.8 ± 18.7	39.0–92.0
Uric acid (U/L)	1.8 ± 0.2	2.6 ± 0.9	0.66–1.60
Urea (U/L)	42.4 ± 1.2	34.4 ± 2.7 *	32.0–54.0

Data are expressed as the mean ± S.E.M. Student’s *t*-test, * *p* < 0.05 (n = 5).

## Data Availability

Data are contained within the article.

## Supplementary Materials

The following supporting information can be downloaded at https://www.mdpi.com/article/10.3390/ph18020221/s1. Figure S1. Original representative recordings of contractile reactivity to KCl in the rat trachea of CG (**A**) and AG (**B**) animals; Figure S2. Representative original recordings of tracheal reactivity induced by 100 µg/mL OVA in CG (**A**), AG (**B**), AAL25G (**C**), AAL50G (**D**), AAL100G (**E**) and ADEXAG (**F**) rats; Figure S3. Original representative recordings of contractile reactivity to CCh from rat trachea of CG (**A**), AG (**B**), AAL25G (**C**), AAL50G (**D**), AAL100G (**E**) and ADEXAG (**F**) rats; Figure S4. Original recordings representing the relaxant reactivity to nifedipine from rat trachea of CG (**A**) and AG (**B**) animals; Figure S5. Original representative recordings of relaxant reactivity to aminophylline from rat trachea of CG (**A**), AG (**B**), AAL25G (**C**), AAL50G (**D**), AAL100G (**E**) and ADEXAG (**F**) animals; Figure S6. 3D interactions of lauric acid (green) under domain-binding conditions with the β_2_-adrenergic receptor (**A**) and binding maps representing the interactions between lauric acid (**B**) and isoprenaline (**C**) with the receptor amino acid residues; Figure S7. 3D interactions of lauric acid (green) under binding domain conditions with Ca_V_ (**A**) and binding maps representing the interactions between lauric acid (**B**) and nifedipine (**C**) with Ca_V_ amino acid residues; Figure S8. 3D interactions of lauric acid (green) under domain-binding conditions with BK_Ca_ (**A**) and binding maps representing the interactions between lauric acid (**B**) and nicorandil (**C**) with BK_Ca_ amino acid residues; Figure S9. 3D interactions of lauric acid (green) under domain-binding conditions with K_ATP_ (**A**) and binding maps representing the interactions between lauric acid (**B**) and glibenclamide (**C**) with K_ATP_ amino acid residues; Figure S10. 3D interactions of lauric acid (green) under domain-binding conditions with adenylyl cyclase (**A**) and binding maps representing the interactions between lauric acid (**B**) and forskolin (**C**) with amino acid residues of adenylyl cyclase; Figure S11. 3D interactions of lauric acid (green) under domain-binding conditions with PKG (**A**) and binding maps representing the interactions between lauric acid (**B**) and cGMP (**C**) with PKG amino acid residues; Figure S12. 3D interactions of lauric acid (green) under domain-binding conditions with eNOS (**A**) and binding maps representing the interactions between lauric acid (**B**) and GW-274150 (**C**) with eNOS amino acid residues; Figure S13. 3D interactions of lauric acid (green) under domain-binding conditions with iNOS (**A**) and binding maps representing the interactions between lauric acid (**B**) and 1400-W (**C**) with iNOS amino acid residues; Figure S14. 3D interactions of lauric acid (green) under binding domain conditions with COX-2 (**A**) and binding maps representing the interactions between lauric acid (**B**) and etoricoxib (**C**) with COX-2 amino acid residues. Table S1. RMSD values for proteins selected in the study; Table S2. Binding energy values (kJ/mol) analyzed in the proteins selected in the study. The best scores in relation to the positive control are highlighted in bold; Table S3. Information on proteins selected in the study; Table S4. Behavioral screening of lauric acid at a dose of 100 mg/kg (p.o.) (Adapted from Almeida et al., 1999). References [121,122,123,124,125,126,127,128,129,130,131,132,133,134,135,136,137,138,139,140,141,142,143,144,145] are citated in the Supplementary Materials.
