# Supplementary material for: Repeated-Dose Toxicity of Lauric Acid and Its Preventive Effect Against Tracheal Hyper-Responsiveness in Wistar Rats with Possible In Silico Molecular Targets
Source: Pharmaceuticals (Basel). 2025 Feb 6;18(2):221. doi: 10.3390/ph18020221 (PMC11859213; doi:10.3390/ph18020221)
Supplement: Supplementary file 1 [file pharmaceuticals-18-00221-s001.zip › pharmaceuticals-3379920-supplementary.pdf]

Article

Repeated-Dose Toxicity of Lauric Acid and Its Preventive Effect Against Tracheal Hyper-responsiveness in Wistar Rats with Possible *In Silico* Molecular Targets

Indyra Alencar Duarte Figueiredo [1,2*], Alissa Maria de Oliveira Martins [1,2], Alexya Mikelle Teixeira Cavalcanti [1], Jayne Muniz Fernandes [1], Ludmila Emilly da Silva Gomes [1], Mateus Mendes Vieira [1], Gabriel Nunes Machado de Oliveira [1], Isabela Motta Felício [1,2], Lucas Nóbrega de Oliveira [1,2], Igor Gabriel da Silva Ramalho [1,2], Natália Ferreira de Sousa [1,2], Luciana Scotti [2], Marcus Tullius Scotti [2,3], José Luiz de Brito Alves [4], Margareth de Fátima Formiga Melo Diniz [2,5], Daniele Idalino Janebro Ximenes [5], Luiz Henrique César Vasconcelos [1,2,6*] and Fabiana de Andrade Cavalcante [1,2,6]

[1] Laboratório de Farmacologia Funcional Prof. George Thomas, Instituto de Pesquisa em Fármacos e Medicamentos, Universidade Federal da Paraíba, João Pessoa 58051-900, PB, Brazil; alissaoliveira@ltf.ufpb.br (A.M.d.O.M.); alexyacavalcanti08@gmail.com (A.M.T.C.); jaynemunizf@gmail.com (J.M.F.); ludmilaesgomes@gmail.com (L.E.d.S.G.); mateusmvd89@gmail.com (M.M.V.); gabrielsoad619@gmail.com (G.N.M.d.O.); isabela_motta@ltf.ufpb.br (I.M.F.); lucasnobrega@ltf.ufpb.br (L.N.d.O.)

[2] Programa de Pós-Graduação em Produtos Naturais e Sintéticos Bioativos, Centro de Ciências da Saúde, Universidade Federal da Paraíba, João Pessoa 58051-900, PB, Brazil; igorgabriel0809@gmail.com (I.G.d.S.R.); nataliafsousa@ltf.ufpb.br (N.F.d.S.); luciana.scotti@gmail.com (L.S.);

[3] Departamento de Química, Centro de Ciências Exatas e da Natureza, Universidade Federal da Paraíba, João Pessoa 58051-900, PB, Brazil; mtscotti@gmail.com (M.T.S.)

[4] Departamento de Nutrição, Centro de Ciências da Saúde, Universidade Federal da Paraíba, João Pessoa 58051-900, PB, Brazil; jose.luiz@academico.ufpb.br

[5] Departamento de Ciências Farmacêuticas, Centro de Ciências da Saúde, Universidade Federal da Paraíba, Cidade Universitária, João Pessoa 58051-900, PB, Brazil; dijanebro@yahoo.com.br; margarethdiniz.ufpb@gmail.com (M.d.F.F.M.D.)

[6] Departamento de Ciências Biomédicas, Centro de Ciências da Saúde, Universidade Federal da Paraíba, Cidade Universitária, João Pessoa 58051-900, PB, Brazil; lhcv@academico.ufpb.br (L.H.C.V.); fabianacavalcante@ltf.ufpb.br (F.d.A.C.)

* Correspondence: indyrafigueiredo@hotmail.com (I.A.D.F.); lhcv@academico.ufpb.br (L.H.C.V.).

Supplementary material

Figure S1 – Original representative recordings of contractile reactivity to KCl in the rat trachea of CG (**A**) and AG (**B**) animals.

(A) **(B)**

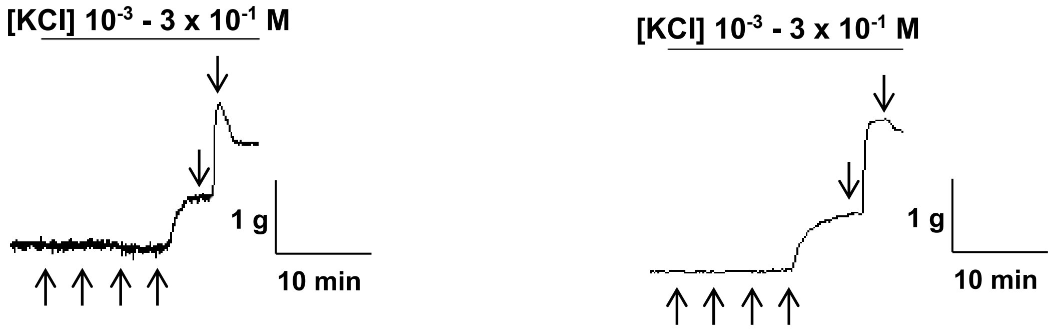

KCl: potassium chloride.

Figure S2 – Representative original recordings of tracheal reactivity induced by 100 µg/mL OVA in CG (**A**), AG (**B**), AAL25G (**C**), AAL50G (**D**), AAL100G (**E**) and ADEXAG (**F**) rats.

(A) **(B)** **(C)** **(D)** **(E)** **(F)**

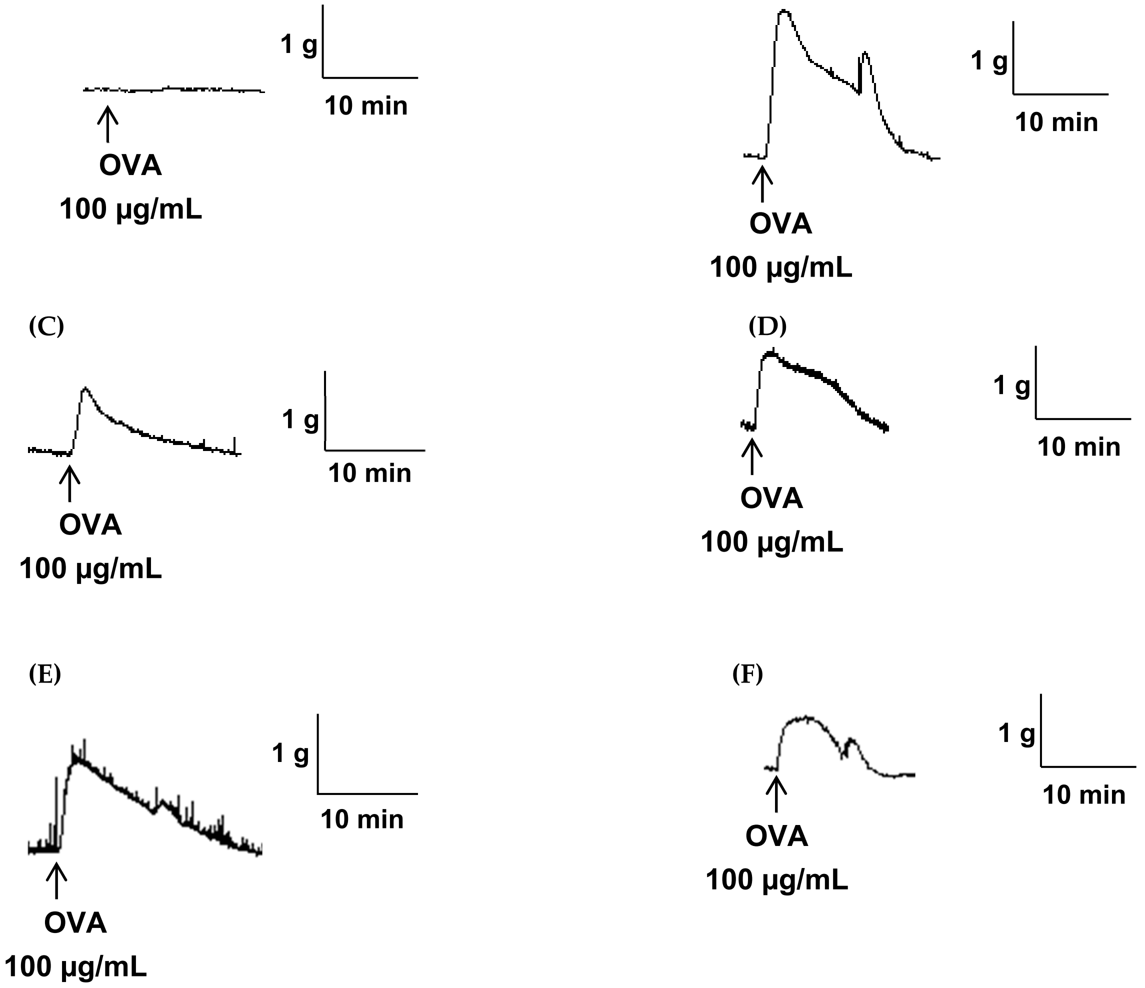

OVA: ovalbumin.

Figure S3 – Original representative recordings of contractile reactivity to CCh from rat trachea of CG (**A**), AG (**B**), AAL25G (**C**), AAL50G (**D**), AAL100G (**E**) and ADEXAG (**F**) rats.

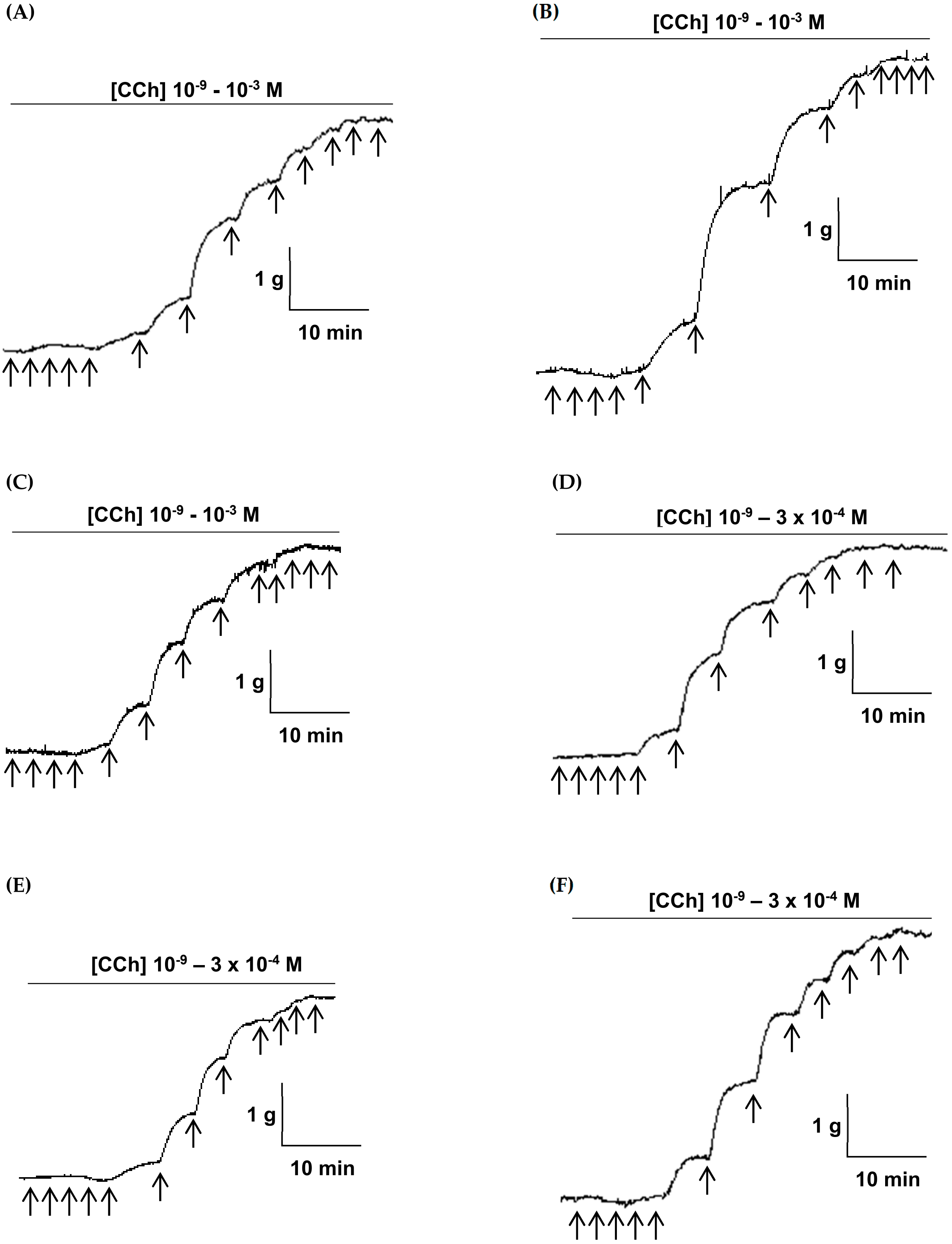

CCh: carbachol.

Figure S4 – Original recordings representing the relaxant reactivity to nifedipine from rat trachea of CG (**A**) and AG (**B**) animals.

(A)

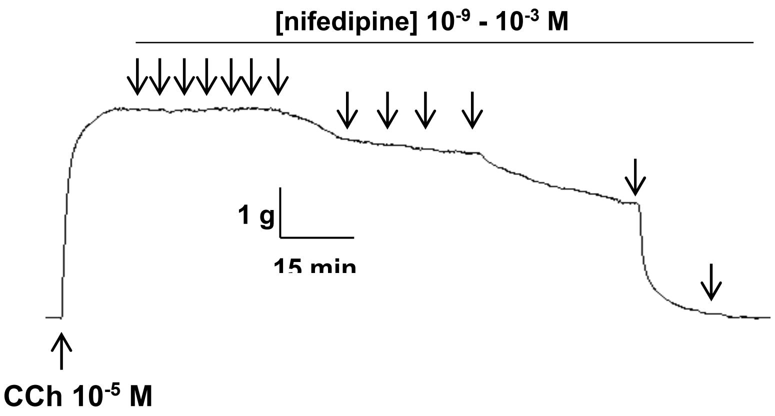

(B)

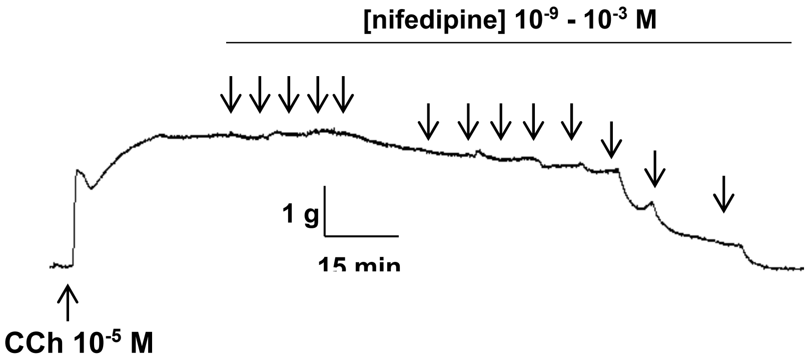

CCh: carbachol.

Figure S5 – Original representative recordings of relaxant reactivity to aminophylline from rat trachea of CG (**A**), AG (**B**), AAL25G (**C**), AAL50G (**D**), AAL100G (**E**) and ADEXAG (**F**) animals.

(A)

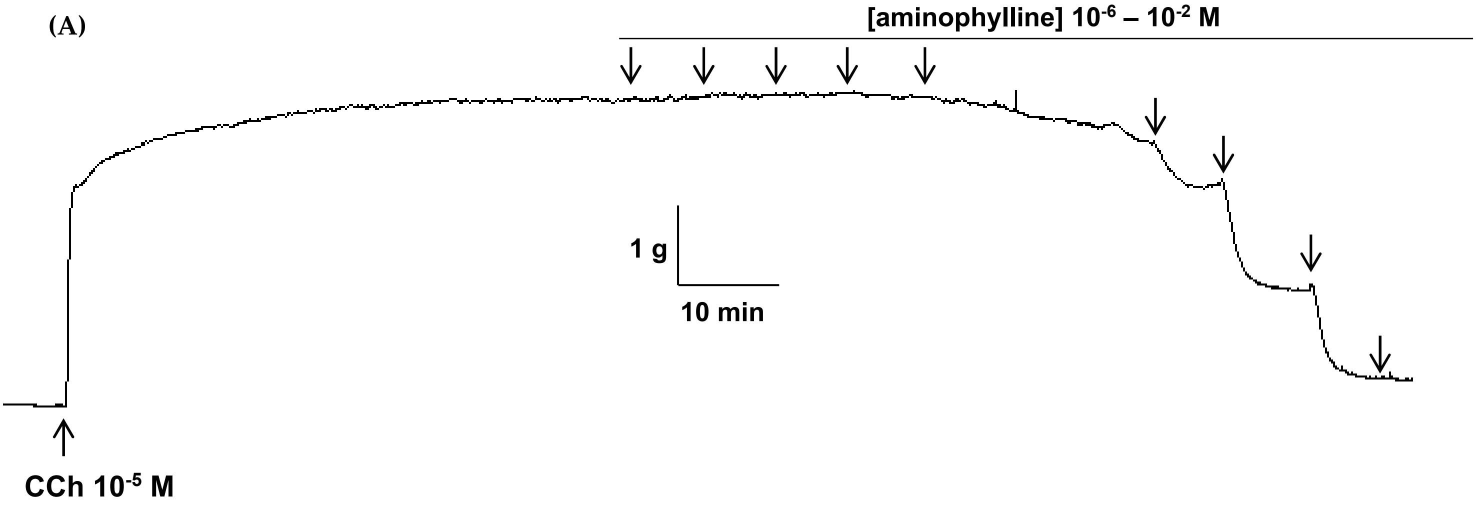

(B)

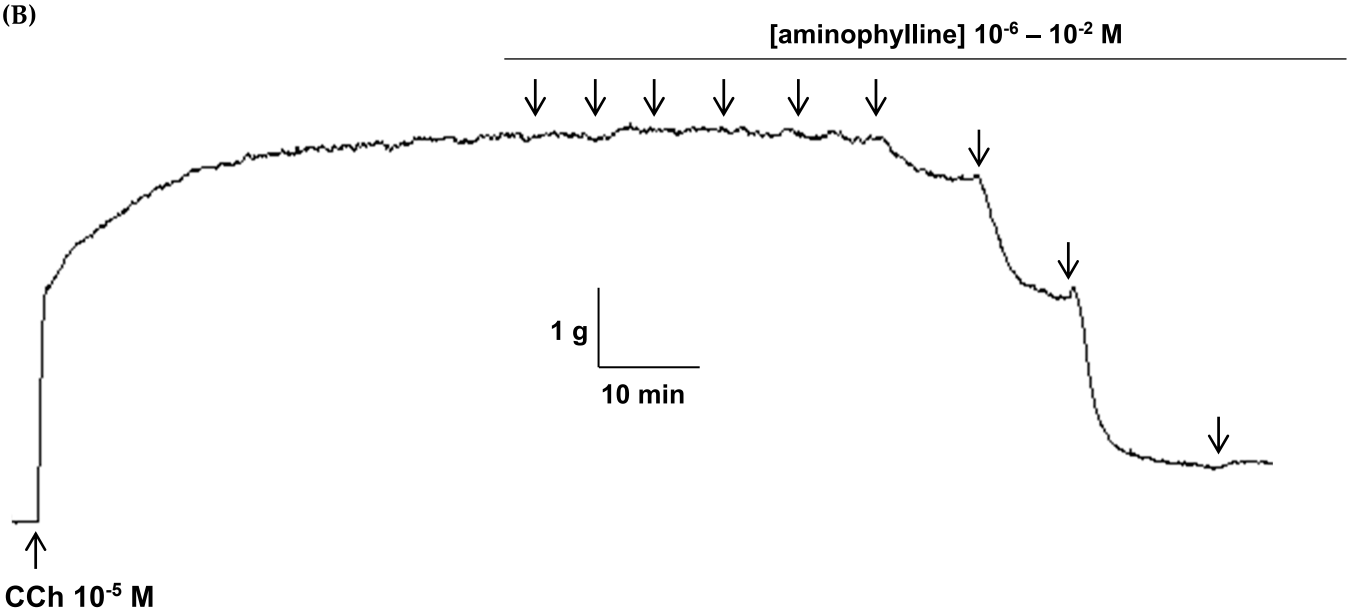

(C)

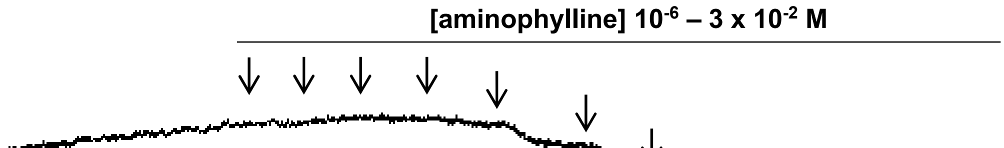

(D)

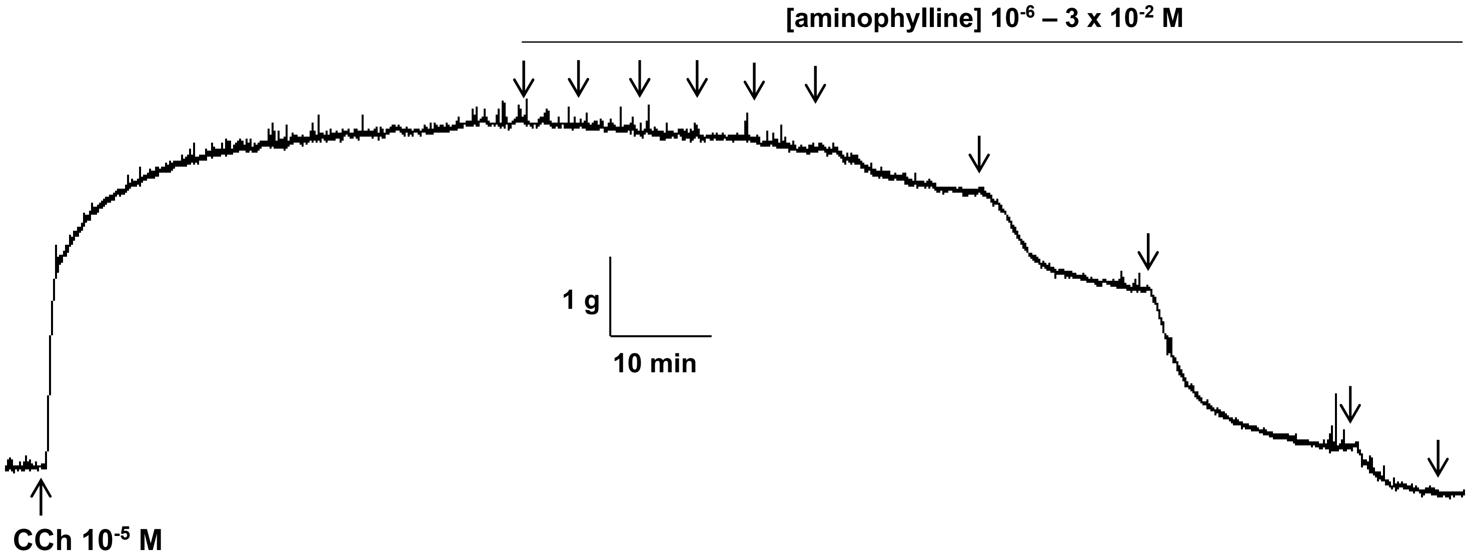

(E)

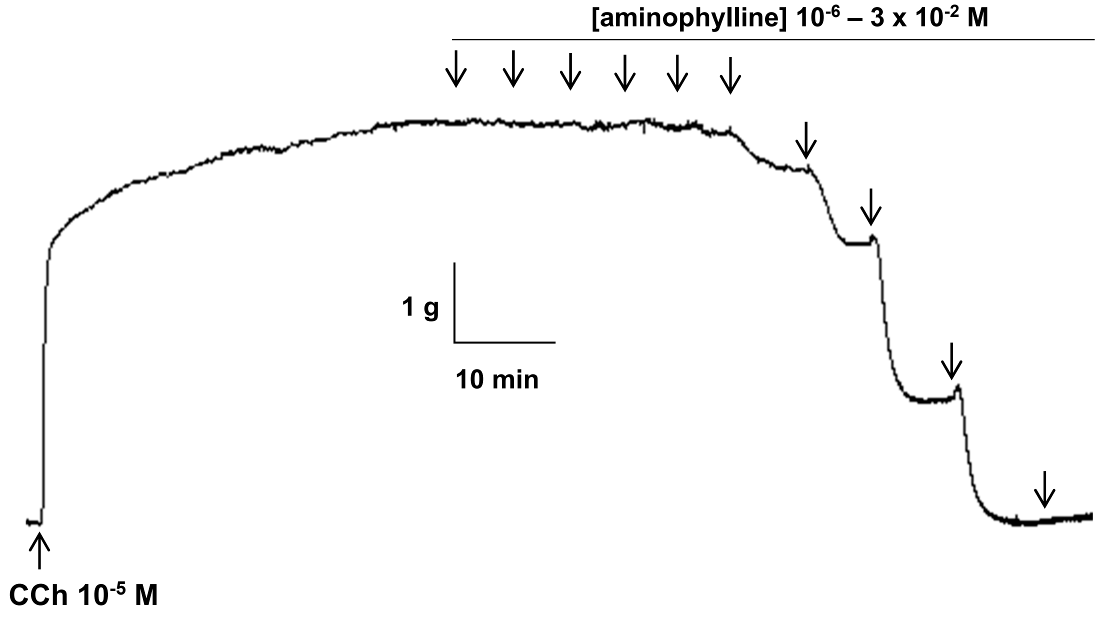

(F)

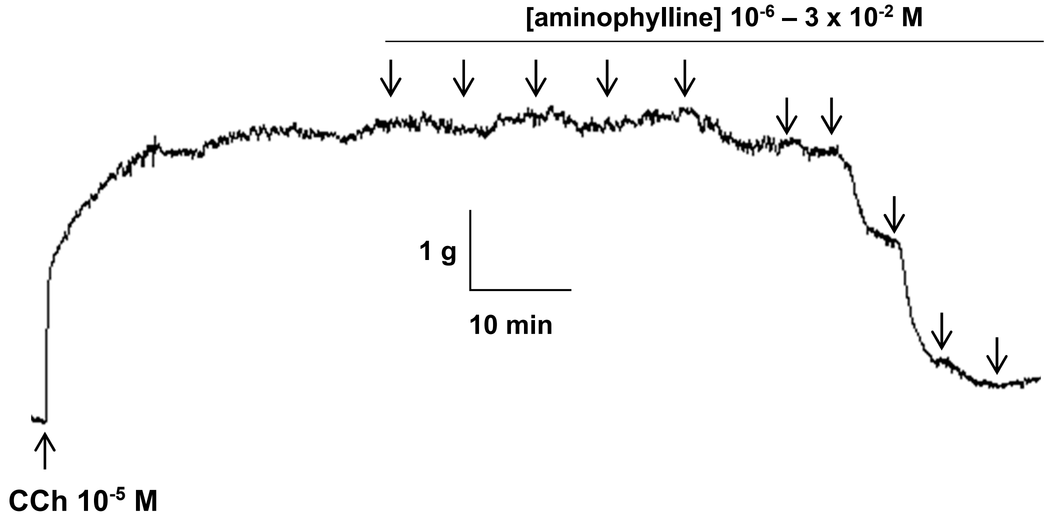

CCh: carbachol.

Table S1 - RMSD values for proteins selected in the study.

Proteins	PDB ID Binder	RMSD
β_2-adrenergic receptor (PDB: 2RH1)	**CAU –** (2S)-1-(9H-Carbazol-4-iloxi)-3-(isopropilamino)propan-2-ol	0,23
H_1 receptor (PDB: 2RZE)	**5EH –** Doxepina (E)	0,56
K_{ATP} (PDB: 6C3P)	**ATP –** Trifosfato de adenosina	0,46
AC (PDB: 4CLZ)	**4DS –** Ácido 4,4'-diisotiociano-2,2'-estilbenodissulfônico	0,19
sCG (PDB: 7D9S)	**YC1 –** [5-[1-(fenilmetil)indazol-3-il]furan-2-il]metanol	0,25
PKA (PDB: 4UJ9)	**S3N –** 7-[(3S,4R)-4-[4-(trifluorometil)fenil]carbonilpirrolidin-3-il]-3H-quinazolin-4-ona	0,30
PKG (PDB: 6BQ8)	**6FW –** 2-amino-8-[(4-clorofenil)sulfanil]-9-[(2S,4aR,6R,7R,7aS)-2,7-dihidroxi-2-oxotetrahidro-2H,4H-2lambda~5~-furo [3,2-d][1,3,2]dioxafosfinin-6-il]-3,9-di-hidro-6H-purin-6-ona	0,70
eNOS (PDB: 1M9J)	**CLW –** Clorzoxazona	0,09
iNOS (PDB: 4NOS)	**H4B –** 5,6,7,8 - Tetrahidrobiopterina	0,20
COX-2 (PDB: 5IKR)	**ID8 –** Ácido mefenâmico	0,14
nNOS (PDB: 5VV5)	**9OJ –** 4-(2-{[(2-amino-4-metilquinolin-7-il)metil]amino}etil)-2-metilbenzonitrila	0,38
ERK-1 (PDB: 4QTB)	**38Z –** (3R)-1-(2-oxo-2-{4-[4-(pirimidin-2-il)fenil]piperazin-1-il}etil)-N-[3-(piridin-4-il)- 2H-indazol-5-il]pirrolidina-3-carboxamida	0,14
ERK-2 (PDB: 6RQ4)	**KE8 –** 6,6-dimetil-2-[2-[(2-metilpirazol-3-il)amino]pirimidin-4-il]-5-(2-morfolin-4-iletil)tieno[2,3-c]pirrol -4-ona	0,22
ROCK-1 (PDB: 2ETR)	**Y27 –** Y-27632	0,17
TGF-β (PDB: 6B8Y)	**D0A** - N-(3-fluoropyridin-4-yl)-2-[6-(trifluoromethyl)pyridin-2-yl]-7H-pyrrolo[2,3-d]pyrimidin-4-amine	0,29

H_1 receptor: histamine receptor 1; Ca_V: voltage-dependent calcium channel; BK_{Ca}: large-conductance calcium-activated potassium channel; K_{ATP}: ATP-sensitive potassium channel; AC: adenylyl cyclase; sCG: soluble guanylyl cyclase; PKA: cAMP-dependent

protein kinase; PKG: cGMP-dependent protein kinase; eNOS: endothelial nitric oxide synthase; iNOS: inducible nitric oxide synthase; nNOS: neuronal nitric oxide synthase; COX-2: cyclooxygenases-2; ERK1/2: extracellular signal-regulated kinases 1 or 2; ROCK-1: Rho-associated protein kinase 1; 5-LOX: lipoxygenase-5; TGF- β: transforming growth factor beta receptor.

Table S2 - Binding energy values (kJ/mol) analyzed in the proteins selected in the study. The best scores in relation to the positive control are highlighted in bold.

Proteins	Lauric acid		Positive control	
	Moldockscore	Rerankscore	Moldockscore	Rerankscore
**β_2-adrenergic receptor (PDB: 2RH1)**	**-98.8103**	**-84.1847**	**-83.772**	**-67.412**
H_1 receptor (PDB: 3RZE)	-112.517	-87.3363	-141.782	-104.695
**Ca_V (PDB: 3G43)**	**-83.2615**	**-60.699**	**-73.8608**	**12.9409**
**BK_{Ca} (PDB: 3AF)**	**-112.247**	**-93.4303**	**-103.044**	**-78.6007**
**K_{ATP} (PDB: 6C3P)**	-92.7847	**-73.4965**	-130.484	**-22.5303**
**AC (PDB: 4CLZ)**	**-67.0243**	**-54.6923**	**-40.7844**	**-35.1598**
sCG (PDB: 7D9S)	-86.1641	-72.5884	-148.771	-77.6362
PKA (PDB: 4UJ9)	-105.561	-84.7153	-121.77	-97.8553
**PKG (PDB: 6BQ8)**	-104.44	**-85.5268**	-120.89	**-37.4022**
**eNOS (PDB: 1N9J)**	**-95.2229**	**-78.9643**	**-79.1829**	**-64.7938**
**iNOS (PDB: 4NOS)**	**-98.2345**	**-83.3349**	**-87.3824**	**-74.6874**
nNOS (PDB: 5VV5)	-48.2912	-39.512	-58.2198	-47.108
**COX-2 (PDB: 5IKR)**	**-97.5696**	**-81.663**	**-101.049**	**45.5035**
ERK-1 (PDB: 4QTB)	-96.5065	-78.8602	-220.281	-192.848
ERK-2 (PDB: 6RQ4)	-66.1648	-53.8009	-153.659	-125.458
ROCK-1 (PDB: 2ETR)	-74.9619	-63.9444	-77.4056	-74.2477
LOX-5	-102.922	-83.7358	-108.331	-84.5529

(PDB: 3O8Y)				
TGF-β (PDB: 6B8Y)	-102.555	-84.7495	-135.327	-104.73

H_1 receptor: histamine receptor 1; Ca_V: voltage-dependent calcium channel; BK_{Ca}: large-conductance calcium-activated potassium channel; K_{ATP}: ATP-sensitive potassium channel; AC: adenylyl cyclase; sCG: soluble guanylyl cyclase; PKA: cAMP-dependent protein kinase; PKG: cGMP-dependent protein kinase; eNOS: endothelial nitric oxide synthase; iNOS: inducible nitric oxide synthase; nNOS: neuronal nitric oxide synthase; COX-2: cyclooxygenases-2; ERK1/2: extracellular signal-regulated kinases 1 or 2; ROCK-1: Rho-associated protein kinase 1; 5-LOX: lipoxygenase-5; TGF- β: transforming growth factor beta receptor.

Table S3 - Information on proteins selected in the study.

Proteins	PDB ID	Positive Control	Resolution
β_2-adrenergic receptor	2RH1 (Cherezov et al., 2007)	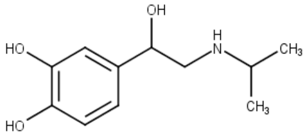 Isoprenalina (Yang et al., 2021)	2.40 Å
H_1 receptor	3RZE (Shimamura et al., 2011)	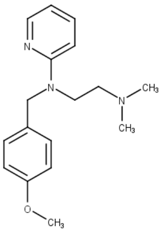 Pirilamina (Levin et al., 2011)	3.10 Å
Ca_V	3G43 (Fallon et al., 2009)	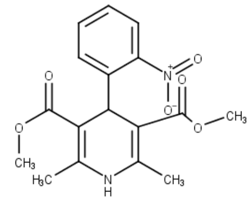 Nifedipino (Spadeto; Freitas; Comanich, 2021),	2.10 Å
BK_{Ca}	3NAF (Wu et al., 2010)	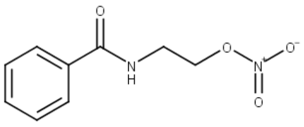 Nicorandil (Evlakhow; Poyasov; Berezina, 2021)	3.10 Å

KATP	6C3P (Lee, Chen Mackinon, 2017)	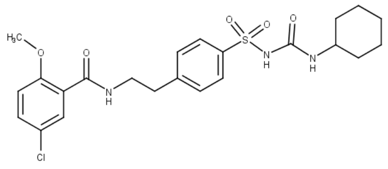 Glibenclamida (Scala et al., 2020)	5.60 Å
AC	4CLZ (Kleinboelting et al., 2014)	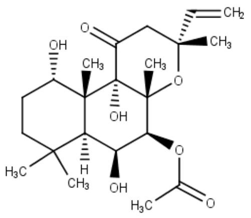 Forscolina (Bhatia et al., 2023)	1.90 Å
sCG	7D9S (Liu; Kang; Chen, 2021)	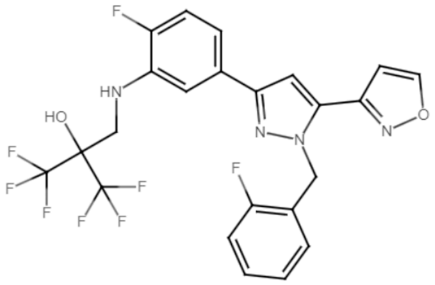 (Hanrahan et al., 2020)	3.90 Å
PKA	4UJ9 (Lauber et al., 2016)	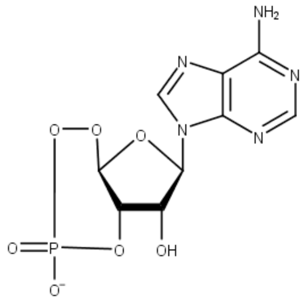 cAMP (Zhang et al., 2020)	1.87 Å
PKG	6BQ8 (Gerlits et al., 2018)	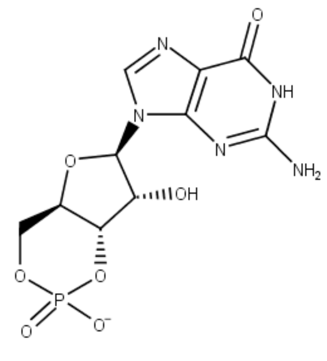 cGMP	2.00 Å

		(Kim et al., 2021)	
eNOS	1M9J<br>(Rosenfeld et al., 2002)	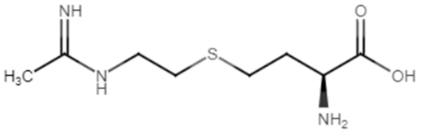<br>GW-274150<br>(Chatterjee et al., 2003)	2.43 Å
iNOS	4NOS<br>(Fischmann et al., 1999)	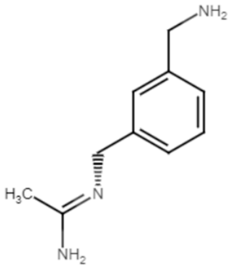<br>1400-W<br>(Jafarian-Therani, 2005)	2.25 Å
nNOS	5VV5<br>(Pensa et al., 2017)	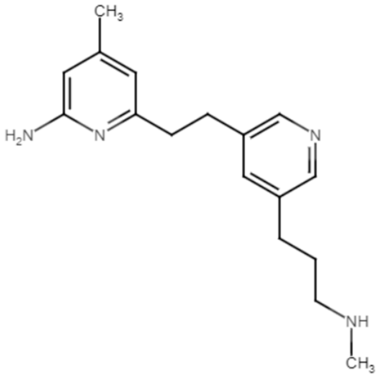<br>4-methyl-6-(2-{5-[3-(methylamino)propyl]pyridin-3-yl}ethyl)pyridin-2-amine<br>(Wang et al., 2016)	2.15 Å
COX-2	5IKR<br>(Orlando; Malkovisk, 2016)	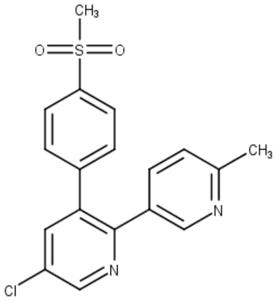<br>Etoricoxibe<br>(Akif et al, 2023)	2.34 Å

LOX-5	3O8Y<br>(Gilbert et al., 2011)	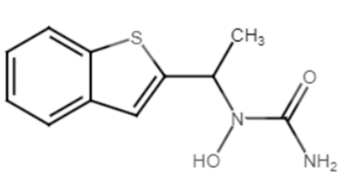<br>Zileutona<br>(Rossi et al., 2010)	2.39 Å
ERK-1	4QTB<br>(Chaikuad et al., 2014)	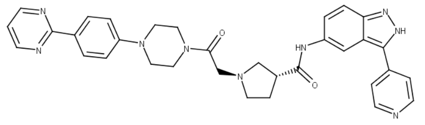<br>(3R)-1-(2-oxo-2-{4-[4-(pyrimidin-2-yl)phenyl]piperazin-1-yl}ethyl)-N-[3-(pyridin-4-yl)-2H-indazol-5-yl]pyrrolidine-3-carboxamide<br>(Chaikuad et al., 2014)	1.40 Å
ERK-2	6RQ4<br>(Kidger et al, 2020)	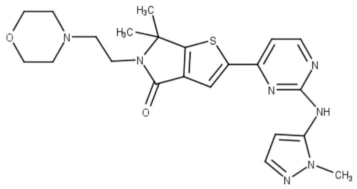<br>6,6-dimethyl-2-[2-[(2-methylpyrazol-3-yl)amino]pyrimidin-4-yl]-5-(2-morpholin-4-ylethyl)thieno[2,3-c]pyrrol-4-one<br>(Kidger et al, 2020)	1.96 Å
ROCK-1	2ETR<br>(Jacobs et al., 2006)	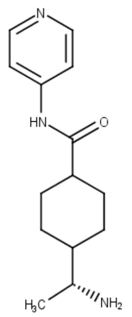<br>Y-27<br>(Jacobs et al., 2006)	2.60 Å
TGF-β receptor	6B8Y<br>(Harikrishnan et al., 2018)	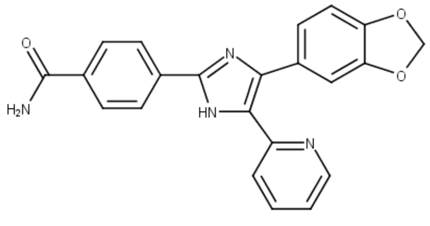	1.65 Å

		SB-431542<br>(Lee et al., 2021)	

H_1 receptor: histamine receptor 1; Ca_V: voltage-dependent calcium channel; BK_{Ca}: large-conductance calcium-activated potassium channel; K_{ATP}: ATP-sensitive potassium channel; AC: adenylyl cyclase; sCG: soluble guanylyl cyclase; PKA: cAMP-dependent protein kinase; PKG: cGMP-dependent protein kinase; eNOS: endothelial nitric oxide synthase; iNOS: inducible nitric oxide synthase; nNOS: neuronal nitric oxide synthase; COX-2: cyclooxygenases-2; ERK1/2: extracellular signal-regulated kinases 1 or 2; ROCK-1: Rho-associated protein kinase 1; 5-LOX: lipoxygenase-5; TGF- β: transforming growth factor beta receptor.

Table S4 - Behavioral screening of lauric acid at a dose of 100 mg/kg (p.o.) (Adapted from Almeida et al., 1999).

**CNS (Central Nervous System) - Stimulant:**	**Until 30 min.**	**1 h**	**2 h**	**3 h**	**4 h**
Aggressiveness					
Increased ambulation					
Circling					
Self-grooming					
Yawning					
Abdominal writhing					
Convulsions					
Climbing					
Stereotypy					
Irritability					
Standing up					
Intense vibrissae movement					
Pedaling					
Head shaking					
Jumping					
Tremors					
Vocalization					
**CNS - Depressant:**	**Until 30 min.**	**1 h**	**2 h**	**3 h**	**4 h**
Hind limb abduction					
Decreased ambulation					
Analgesia					
Anesthesia					
Ataxia					
Catatonia					
Straub tail					
Hypnosis					
Loss of auricular reflex					
Loss of corneal reflex					
Eyelid ptosis					
Righting reflex					

Decreased touch response					
Sedation					
**ANS (Autonomic Nervous System):**	**Until 30 min.**	**1 h**	**2 h**	**3 h**	**4 h**
Cyanosis					
Constipation					
Defecation					
Diarrhea					
Grip strength					
Lacrimation (tearing)					
Urination					
Piloerection (hair standing on end)					
Respiration					
Salivation					
Muscle tone					
**DEATH**					

Figure S6 - 3D interactions of lauric acid (green) under domain-binding conditions with the β_2-adrenergic receptor (**A**) and binding maps representing the interactions between lauric acid (**B**) and isoprenaline (**C**) with the receptor amino acid residues. Hydrophobic interactions (pink dotted lines); hydrogen bonds (green dotted lines); oxygen atoms (red); amino acid residues: Asn = asparagine; Asp = aspartic acid; Phe = phenylalanine; Ser = serine; Val = valine.

(A)

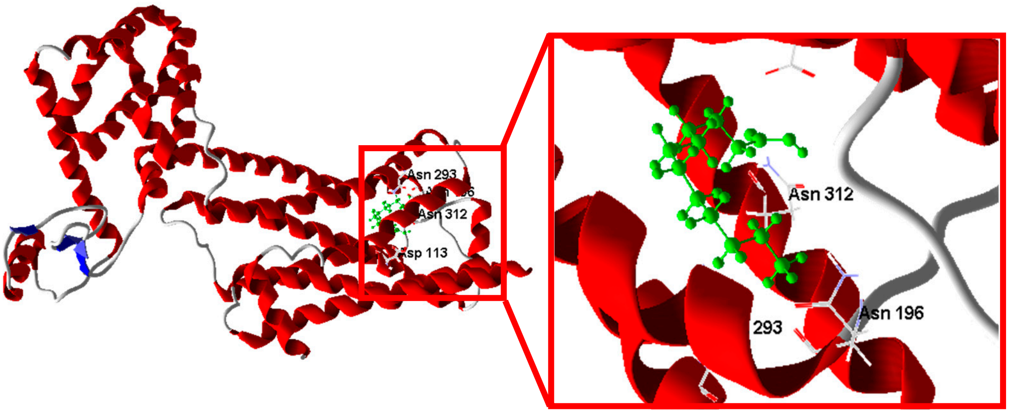

(B)

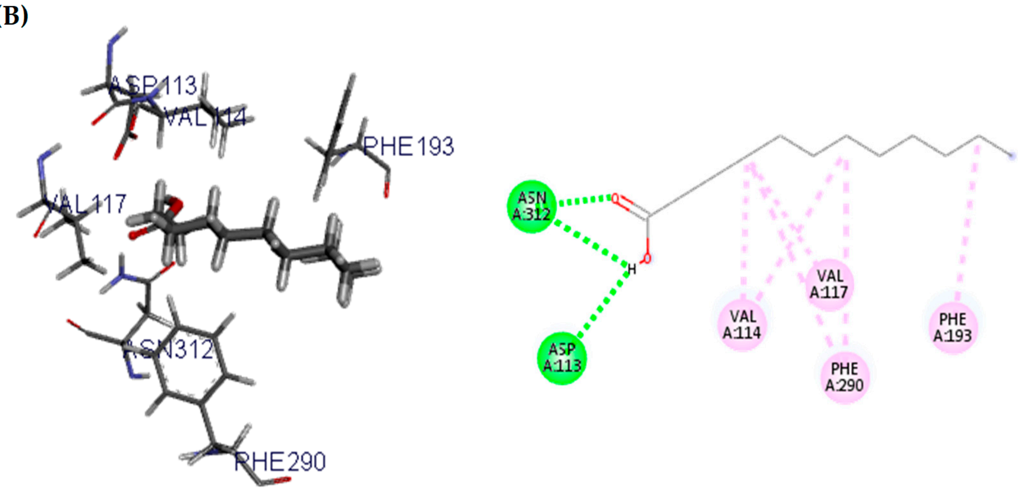

(C)

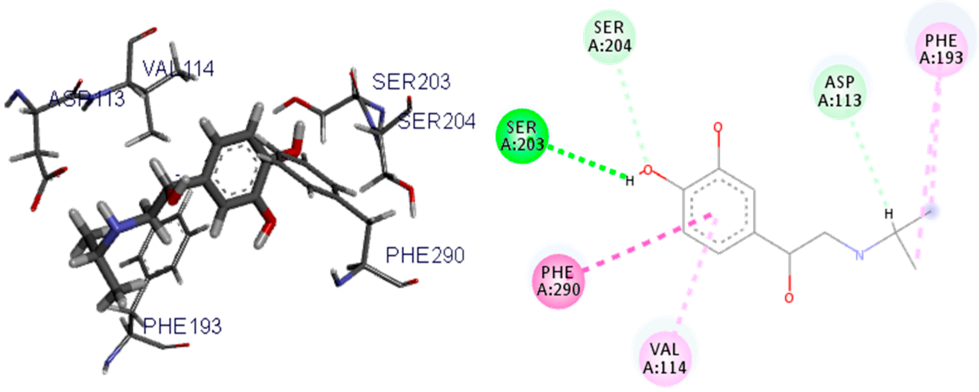

Figure S7 - 3D interactions of lauric acid (green) under binding domain conditions with Cav (**A**) and binding maps representing the interactions between lauric acid (**B**) and nifedipine (**C**) with Cav amino acid residues. Hydrophobic interactions (pink dotted lines); steric interactions (red dotted lines); hydrogen bonds (green dotted lines); oxygen atoms (red); amino acid residues: Ala = alanine; Arg = arginine; Glu = glutamic acid; Met = methionine; Val = valine.

(A)

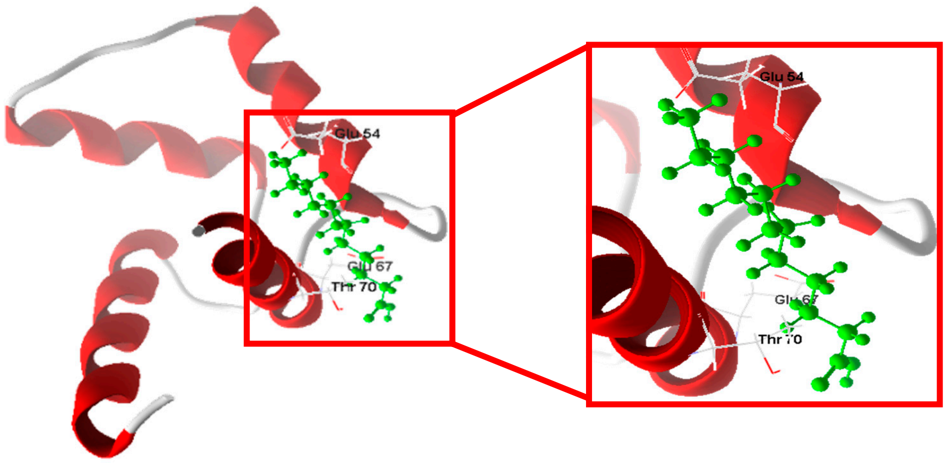

(B)

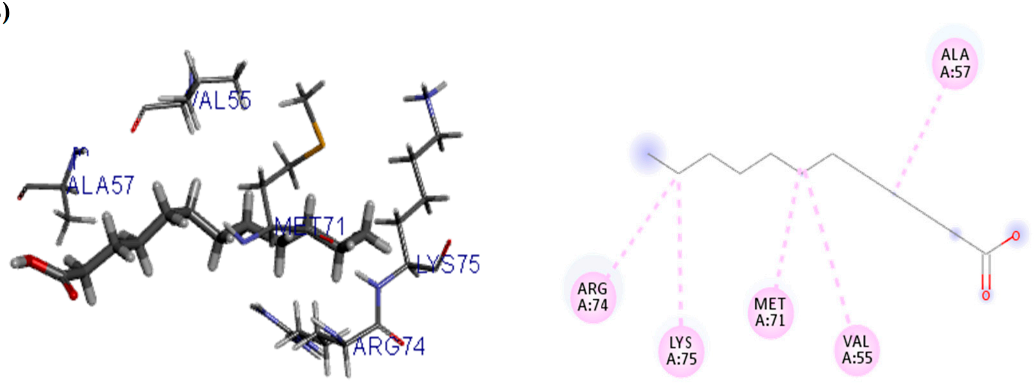

(C)

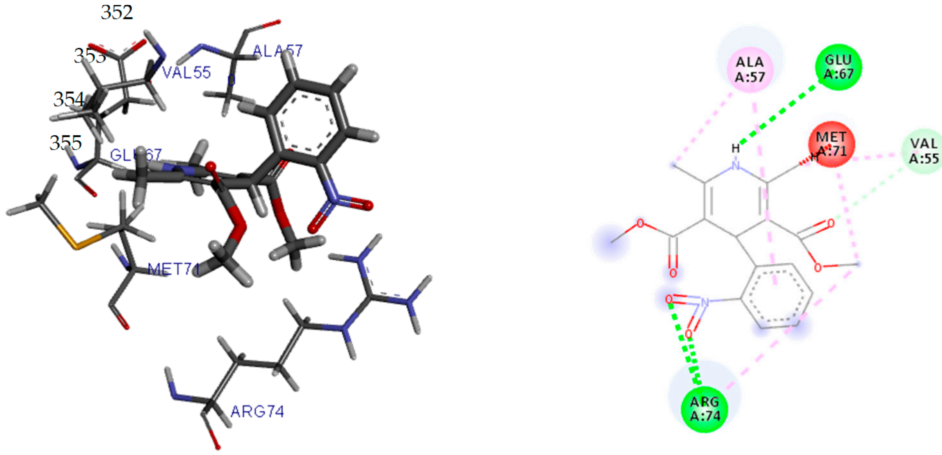

Figure S8 - 3D interactions of lauric acid (green) under domain-binding conditions with BK_{Ca} (**A**) and binding maps representing the interactions between lauric acid (**B**) and nicorandil (**C**) with BK_{Ca} amino acid residues. Hydrophobic interactions (pink dotted lines); steric interactions (red dotted lines); hydrogen bonds (green dotted lines); oxygen atoms (red); amino acid residues: Asn = asparagine; Cys = cysteine; Gly = glycine; Gln = glutamine; Met = methionine; Ser = serine; Thr = threonine; Trp = tryptophan; Val = valine.

(A)

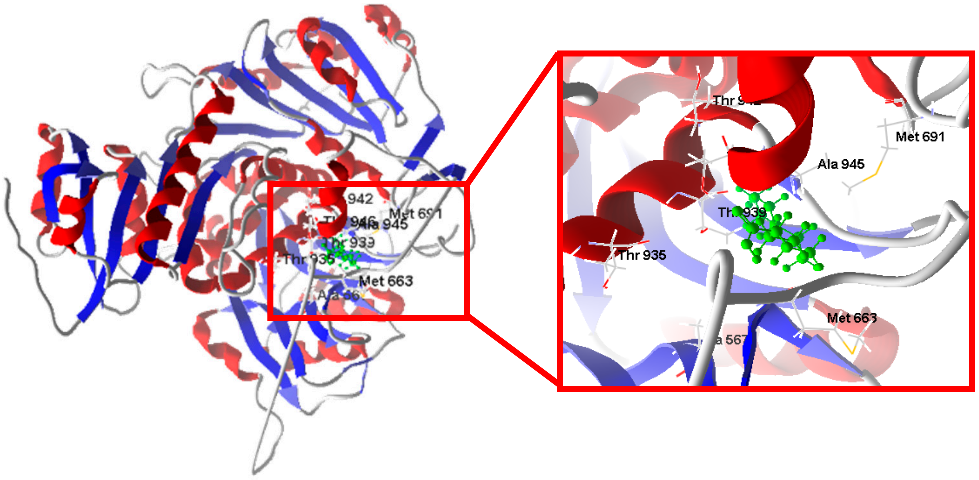

(B)

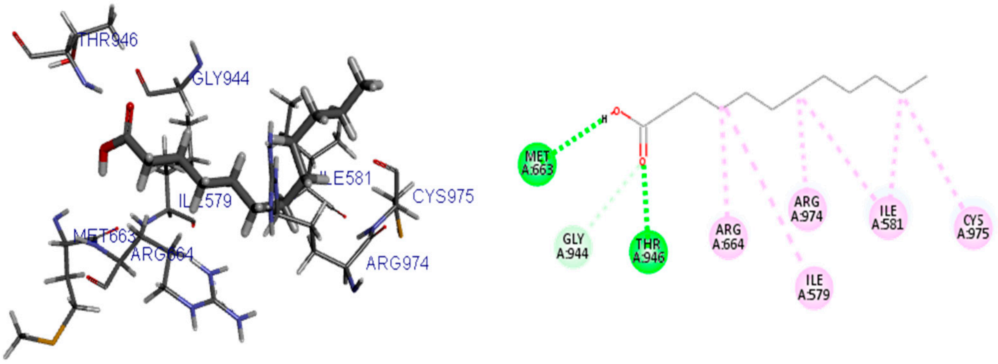

(C)

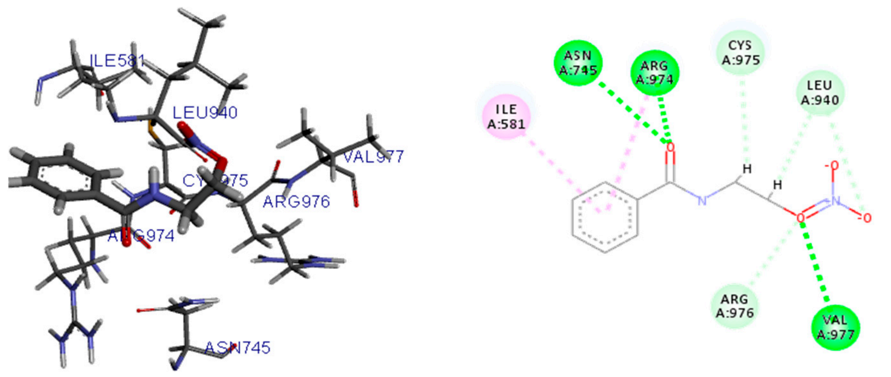

Figure S9 - 3D interactions of lauric acid (green) under domain-binding conditions with K_{ATP} (**A**) and binding maps representing the interactions between lauric acid (**B**) and glibenclamide (**C**) with K_{ATP} amino acid residues. Hydrophobic interactions (pink dotted lines); steric interactions (red dotted lines); hydrogen bonds (green dotted lines); oxygen atoms (red); amino acid residues: Asn = asparagine; Gly = glycine; Gln = glutamine; Met = methionine; Ser = serine; Trp = tryptophan; Val = valine.

(A)

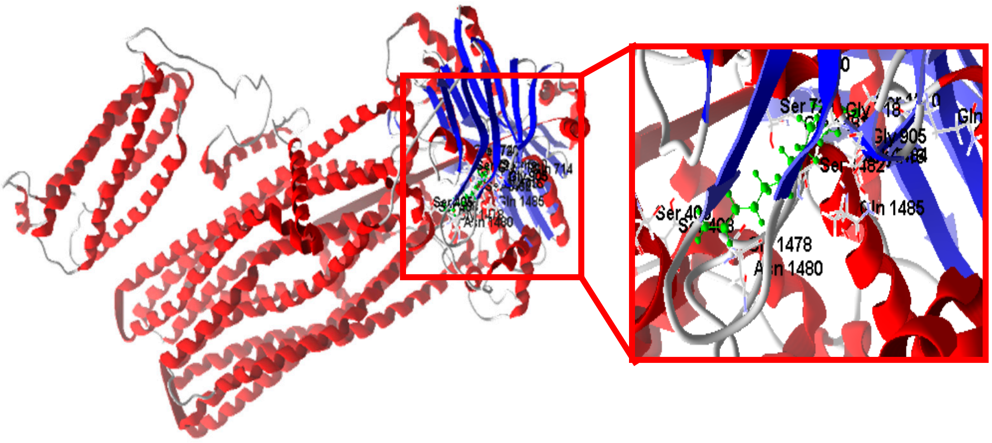

(B)

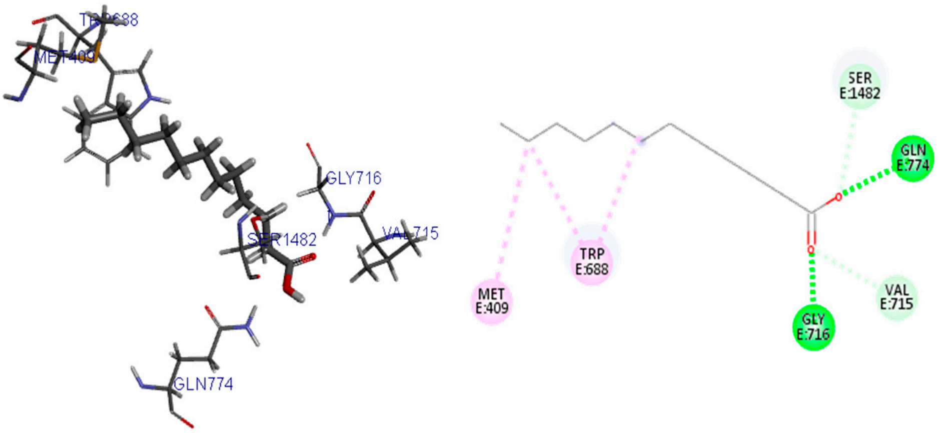

(C)

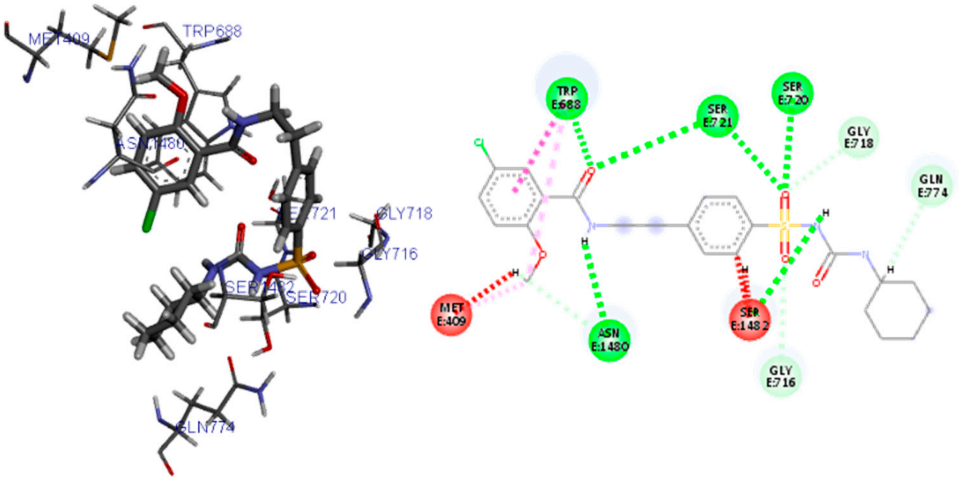

Figure S10 - 3D interactions of lauric acid (green) under domain-binding conditions with adenylyl cyclase (**A**) and binding maps representing the interactions between lauric acid (**B**) and forskolin (**C**) with amino acid residues of adenylyl cyclase. Hydrophobic interactions (pink dotted lines); steric interactions (red dotted lines); hydrogen bonds (blue dotted lines); oxygen atoms (red); amino acid residues: Ala = alanine; Arg = arginine; Asp = aspartic acid; Phe = phenylalanine; Val = valine.

(A)

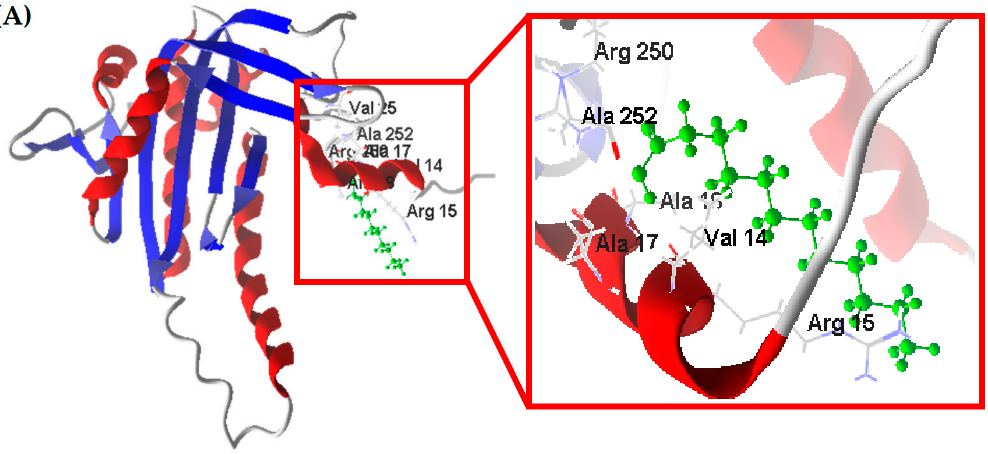

(B)

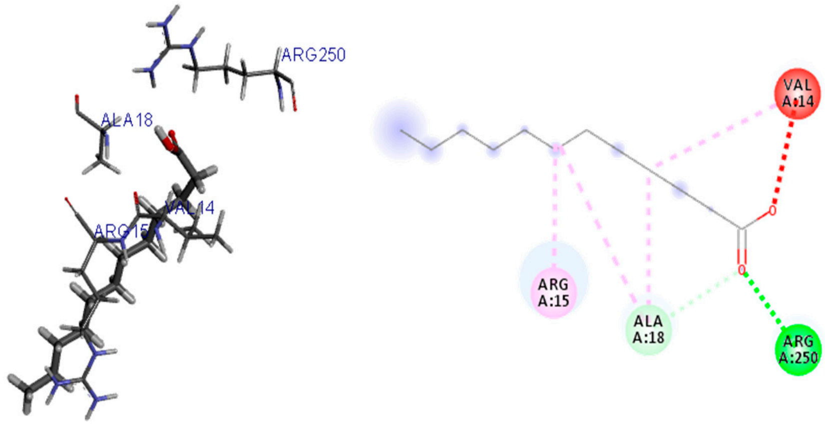

(C)

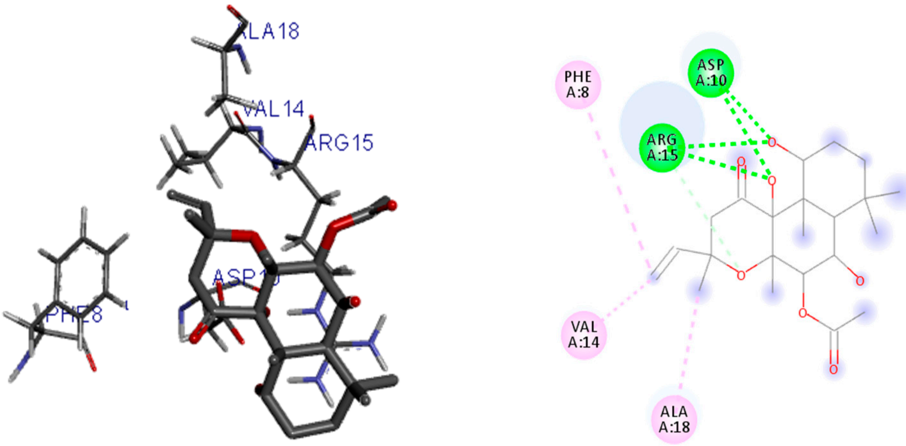

Figure S11 – 3D interactions of lauric acid (green) under domain-binding conditions with PKG (**A**) and binding maps representing the interactions between lauric acid (**B**) and cGMP (**C**) with PKG amino acid residues. Hydrophobic interactions (pink dotted lines); steric interactions (red dotted lines); hydrogen bonds (green dotted lines); oxygen atoms (red); amino acid residues: Ala = alanine; Arg = arginine; Asp = aspartate; Gln = glutamine; Gly = glycine; Leu = leucine; Lys = lysine; Ser = serine; Val = valine.

(A)

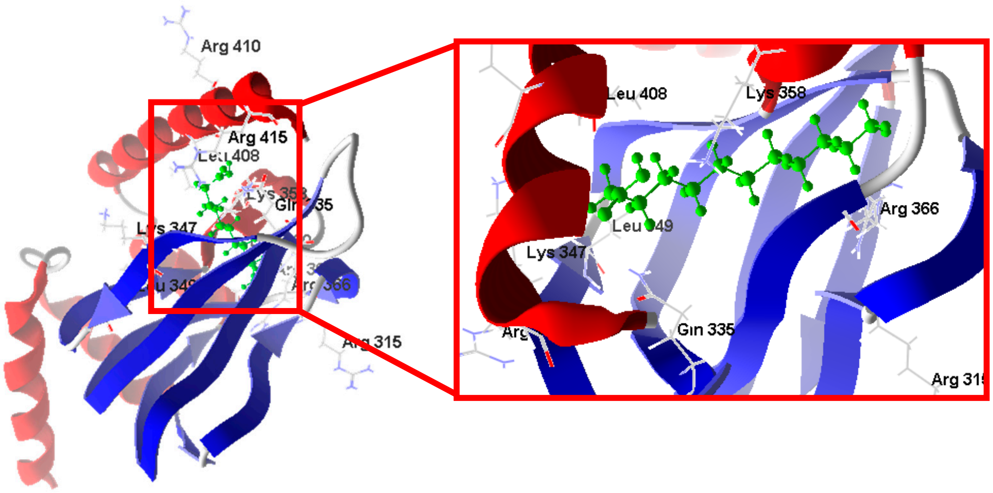

(B)

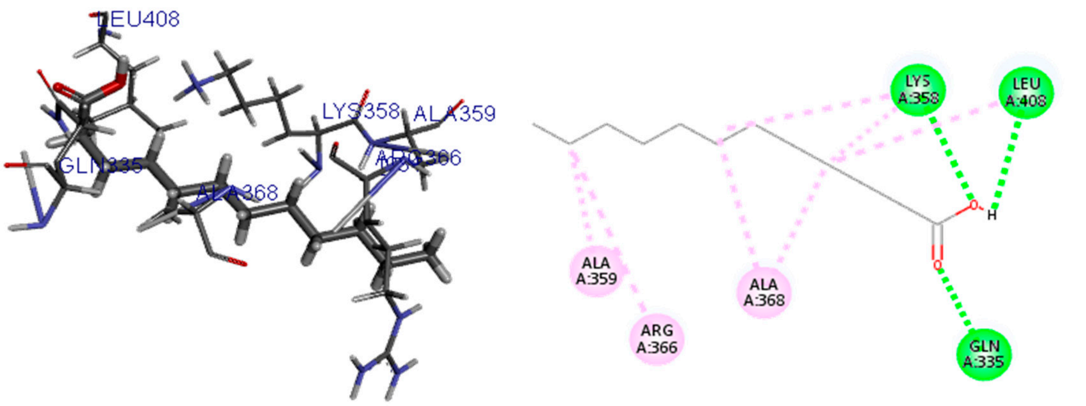

(C)

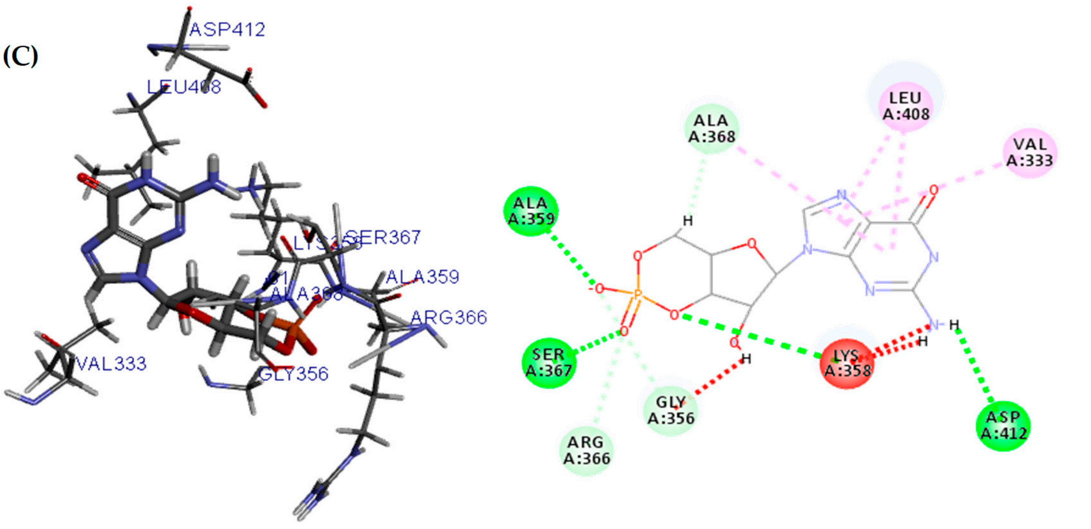

Figure S12 - 3D interactions of lauric acid (green) under domain-binding conditions with eNOS (**A**) and binding maps representing the interactions between lauric acid (**B**) and GW-274150 (**C**) with eNOS amino acid residues. Hydrophobic interactions (pink dotted lines); hydrogen bonds (green dotted lines); steric interactions (orange dotted lines); oxygen atoms (red); amino acid residues: Phe = phenylalanine; Pro = proline; Trp = Tryptophan; Leu = Leucine; Phe = phenylalanine.

(A)

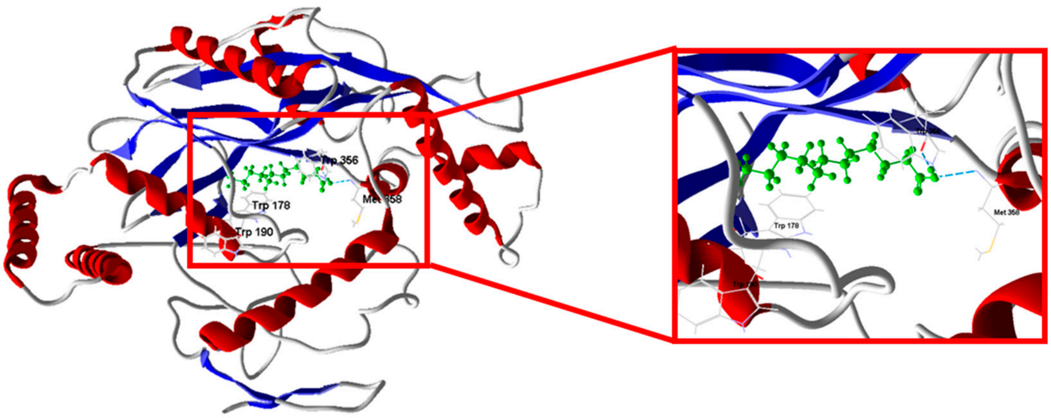

(B)

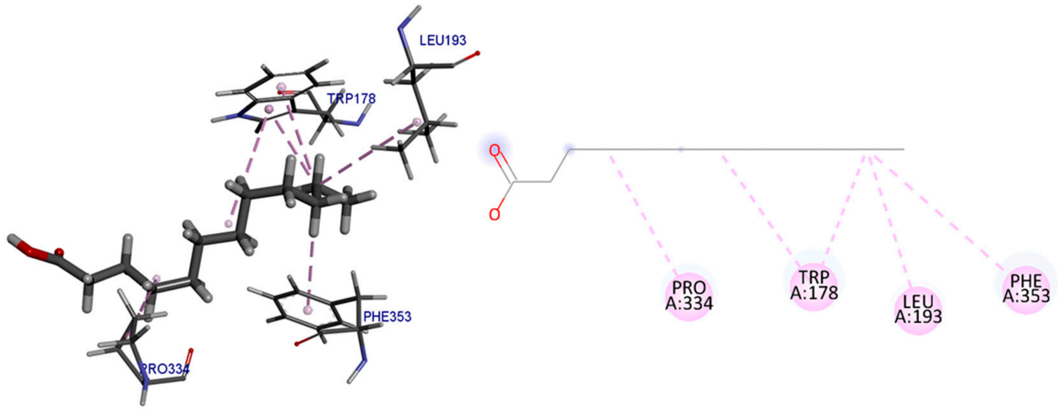

(C)

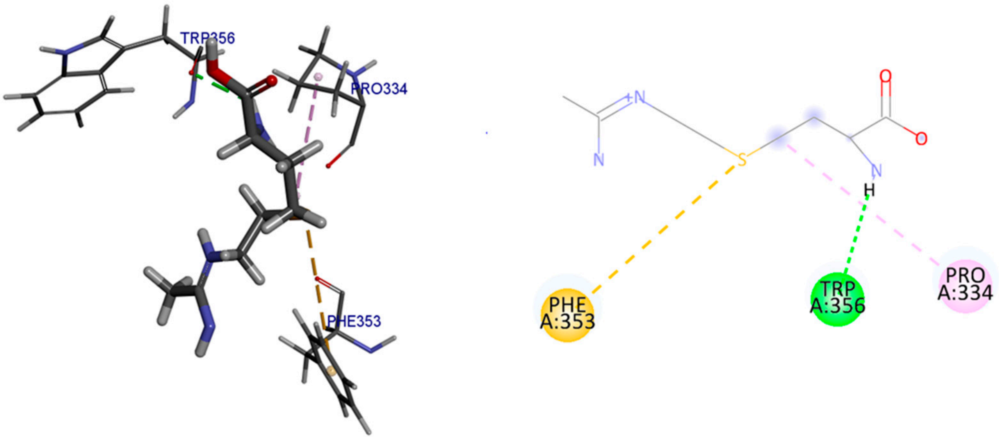

Figure S13 – 3D interactions of lauric acid (green) under domain-binding conditions with iNOS (**A**) and binding maps representing the interactions between lauric acid (**B**) and 1400-W (**C**) with iNOS amino acid residues. Hydrophobic interactions (pink dotted lines); steric interactions (orange dotted lines); hydrogen bonds (green dotted lines); oxygen atoms (red); amino acid residues: Arg = arginine; Ile = isoleucine; Met = methionine; Trp = tryptophan; Phe = phenylalanine.

(A)

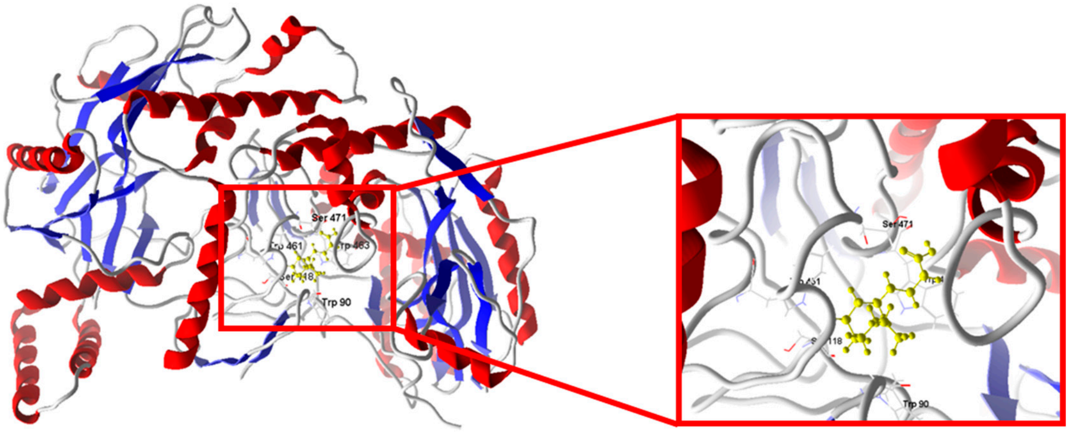

(B)

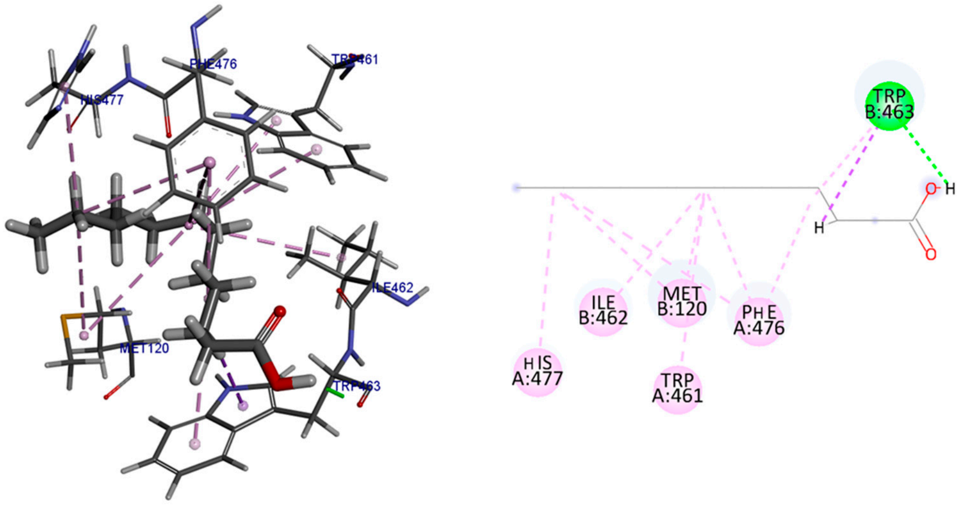

(C)

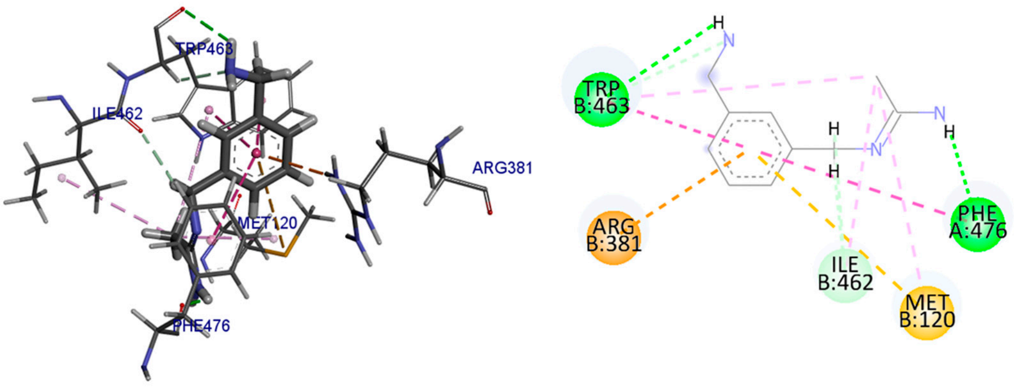

Figure S14 – 3D interactions of lauric acid (green) under binding domain conditions with COX-2 (**A**) and binding maps representing the interactions between lauric acid (**B**) and etoricoxib (**C**) with COX-2 amino acid residues. Hydrophobic interactions (pink dotted lines); steric interactions (red and orange dotted lines); hydrogen bonds (green dotted lines); oxygen atoms (red); amino acid residues: Ala = alanine; Leu = leucine; Met = methionine; Phe = phenylalanine; Ser = serine; Trp = tryptophan; Tyr = tyrosine; Val = valine.

(A)

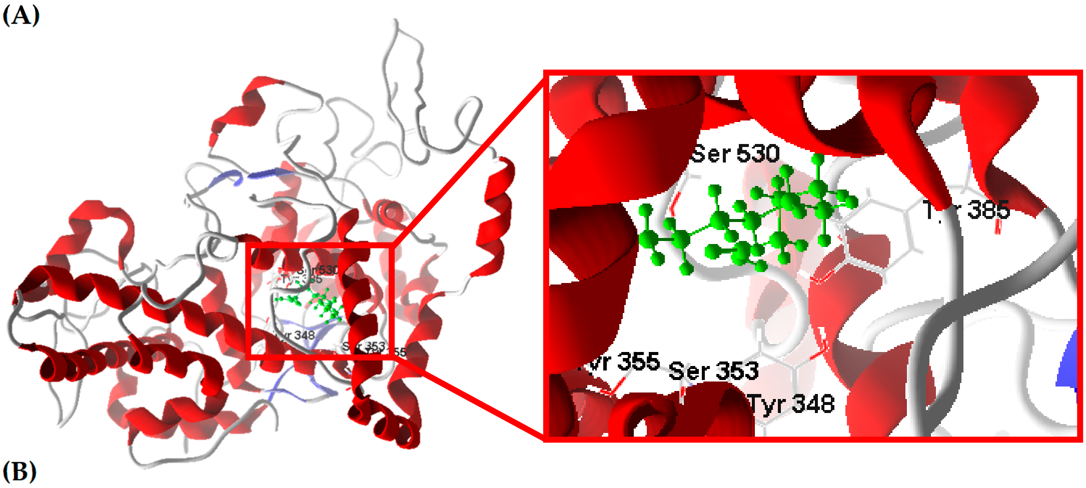

(B)

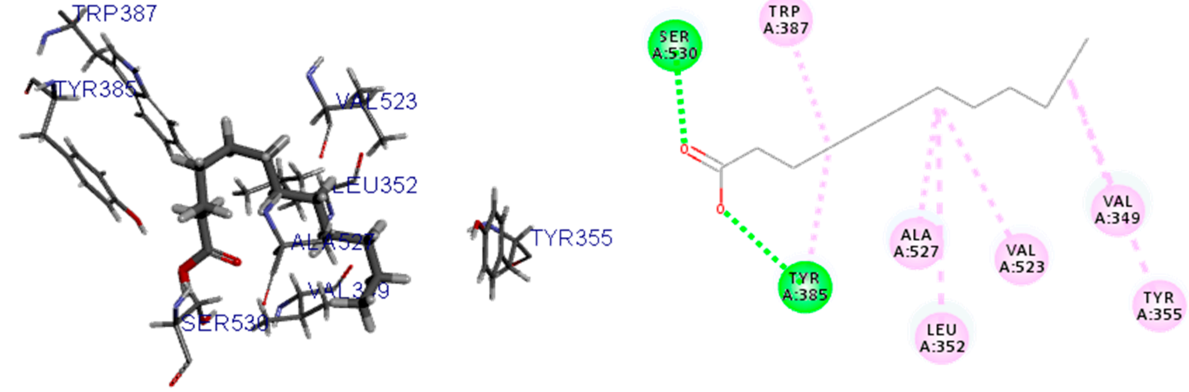

(C)

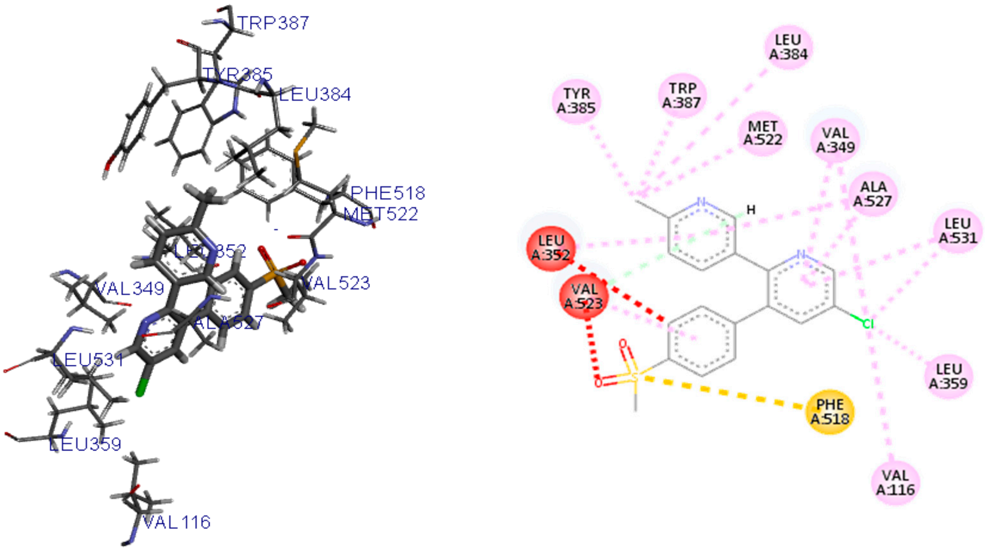

References

Akif UM, Miyan J, Rana R, Moinuddin, Goswami NK, Tanzeela, et al. Selective COX-2 inhibitor etoricoxib's liposomal formulation attenuates M2 polarization of TAMs and enhances its anti-metastatic potential. Pharm Res. 2023;40(2):551-566. 10.1007/s11095-023-03491-z

Bhatia V, Maghsoudi S, Hinton M, Bhagirath AY, Singh N, Jaggupilli A, et al. Characterization of adenylyl cyclase isoform 6 residues interacting with forskolin. Biology. 2023;12(4):572. 10.3390/biology12040572.

Chaikuad A, Tacconi EMC, Zimmer J, Liang Y, Gray NS, Tarsounas M. A unique inhibitor binding site in ERK1/2 is associated with slow binding kinetics. Nat Chem Biol. 2014;10(10):853-860. 10.1038/nchembio.1629

Chatterjee PK, Patel NS, Sivarajah A, Kvale EO, Dugo L, Cuzzocrea, S, et al. GW274150, a potent and highly selective inhibitor of iNOS, reduces experimental renal ischemia/reperfusion injury. Kidney Int. 2003;63(3):853-865. 10.1046/j.1523-1755.2003.00703.x

Cherezov V, Rosenbaum DM, Hanson MA, Rasmussen SGF, Thian FS, Kobilka TS. High-resolution crystal structure of an engineered human β_2-adrenergic G protein–coupled receptor. Science. 2007;318:1258–1265. 10.1126/science.1150577

Evlakhov VI, Poyasov IZ, Berezina TP. Changes of pulmonary microhemodynamics in experimental pulmonary thromboembolism after pretreatment with K-channel activators. Bull Exp Biol Med. 2022;173(3), 302-305. 10.1007/s10517-022-05802-3

Fallon JL, Baker MR, Xiong L, Loy RE, Yang G, Dirksen RT, et al. Crystal structure of dimeric cardiac L-type calcium channel regulatory domains bridged by Ca^{2+}-calmodulins. Proc Natl Acad Sci. 2009;106:5135–5140. 10.1073/pnas.0807487106

Fischmann TO, Hruza A, Niu XD, Fossetta JD, Lunn CA, Dolphin E, et al. Structural characterization of nitric oxide synthase isoforms reveals striking active-site conservation. Nat Struct Biol. 1999;6(3):233-242. 10.1038/6675.

Gerlits O, Campbell JC, Blakeley MP, Kim C, Kovalevsky A. Neutron Crystallography Detects Differences in Protein Dynamics: Structure of the PKG II Cyclic Nucleotide Binding Domain in Complex with an Activator. Biochemistry. 2018: 57, 1833–1837. 10.1021/acs.biochem.8b00010.

Gilbert NC, Bartlett SG, Waight MT, Neau DB, Boeglin WE, Brash AR, et al. The structure of human 5-lipoxygenase. Science. 2011;331(6014):217-219. 10.1126/science.1198482

Hanrahan JP, de Boer IH, Bakris GL, Wilson PJ, Wakefield JD, Seferovic JP, et al. Effects of the soluble guanylate cyclase stimulator praliciguat in diabetic kidney disease: A randomized placebo-controlled clinical trial. Clin J Am Soc Nephrol. 2021;16(1):59-69. 10.2215/CJN.03450320

Harikrishnan LS, Warrier J, Tebben AJ, Tonukunuru G, Madduri SR, Baligar V, et al. Heterobicyclic inhibitors of transforming growth factor beta receptor I (TGFβRI). Bioorg Med Chem. 2018;26(5):1026-1034. 10.1016/j.bmc.2018.01.014

Jacobs M, Hayakawa K, Swenson L, Bellon S, Fleming M, Taslimi P, et al. The structure of dimeric ROCK I reveals the mechanism for ligand selectivity. J Biol Chem. 2006;281:260–268. 10.1074/jbc.M508847200

Jafarian-Tehrani M, Louin G, Royo NC, Besson VC, Bohme GA, Plotkine M, et al. 1400W, a potent selective inducible NOS inhibitor, improves histopathological outcome following traumatic brain injury in rats. Nitric Oxide. 2005;12(2):61-69. 10.1016/j.niox.2005.01.001

Kidger AM, Munck JM, Saini HK, Balmanno K, Minihane E, Courtin A, et al. Dual-mechanism ERK1/2 inhibitors exploit a distinct binding mode to block phosphorylation and nuclear accumulation of ERK1/2. Mol. Cancer Ther. 2020;19:525–

539. 10.1158/1535-7163

Kim SM, Yuen T, Iqbal J, Rubin MR, Zaidi M. The NO–cGMP–PKG pathway in skeletal remodeling. Ann N Y Acad Sci. 2021;1487(1):21-30. 10.1111/nyas.14645

Kleinboelting S, Diaz A, Moniot S, van den Heuvel J, Weyand M, Levin LR, et al. Crystal structures of human soluble adenylyl cyclase reveal mechanisms of catalysis and of its activation through bicarbonate. Proc Natl Acad Sci. 2014;111:3727–3732. 10.1073/pnas.1322778111.

Lauber BS, Hardegger LA, Asraful AK, Lund BA, Dumele O, Harder M, et al. Addressing the glycine-rich loop of protein kinases by a multi-facetted interaction network: inhibition of PKA and a PKB mimic. Chem Eur J. 2016;22:211–221. 10.1002/chem.201503552

Lee AJ, Mahoney CM, Cai CC, Ichinose R, Stefani RM, Marra KG, et al. Sustained delivery of SB-431542, a type I transforming growth factor beta-1 receptor inhibitor, to prevent arthrofibrosis. Tissue Eng Part A. 2021;27(21-22):1411-1421. 10.1089/ten.tea.2021.0204

Lee KPK, Chen J, MacKinnon R. Molecular structure of human K_{ATP} in complex with ATP and ADP. Elife. 2017;6:e32481. 10.7554/eLife.32481

Levin ED, Slade S, Wells C, Pruitt M, Cousins V, Cauley M, et al. Histamine H1 antagonist treatment with pyrilamine reduces nicotine self-administration in rats. Eur J Pharmacol. 2011;650(1):256-260. 10.1016/j.ejphar.2010.10.012

Liu R, Kang Y, Chen L. Activation mechanism of human soluble guanylate cyclase by stimulators and activators. Nat Commun. 2021;12:1–10. 10.1038/s41467-021-25617-0.

Pensa AV, Cinelli MA, Li H, Chreifi G, Mukherjee P, Roman LJ, et al. Hydrophilic, potent, and selective 7-substituted 2-aminoquinolines as improved human neuronal nitric oxide synthase inhibitors. J Med Chem, 2017;60:7146–7165. 10.1021/acs.jmedchem.7b00835.

Rosenfeld RJ, Garcin ED, Panda K, Andersson G, Aberg A, Wallace AV, et al. Conformational changes in nitric oxide synthases induced by chlorzoxazone and nitroindazoles: Crystallographic and computational analyses of inhibitor potency. Biochemistry. 2002;41(46):13915–13925. 10.1021/bi026313j.

Rossi A, Pergola C, Koeberle A, Hoffmann M, Dehm F, Bramanti P, et al. The 5-lipoxygenase inhibitor, zileuton, suppresses prostaglandin biosynthesis by inhibition of arachidonic acid release in macrophages. Br J Pharmacol. 2010;161(3):555-570. 10.1111/j.1476-5381.2010.00861.x

Scala R, Maqoud F, Zizzo N, Mele A., Camerino GM, Zito FA, et al. Pathophysiological consequences of K_{ATP} channel overactivity and pharmacological response to glibenclamide in skeletal muscle of a murine model of Cantù syndrome. Front Pharmacol. 2020;11:604885. 10.3389/fphar.2020.604885

Shimamura T, Shiroishi M, Weyand S, Tsujimoto H, Winter G, Katritch V, Abagyan R, Cherezov V, Liu W, Han GW. Structure of the human histamine H1 receptor complex with doxepin. Nature. 2011;475: 65–70. 10.1038/nature10236.

Spadeto JPM, Freitas MP, Cormanich RA. Fluorinated dihydropyridines as candidates to block L-type voltage-dependent calcium channels. J Biomol Struct Dyn. 2022;40(24):13456-13471. 10.1080/07391102.2021.1963620

Wang HY, Qin Y, Li H, Roman LJ, Martásek P, Poulos TL, et al. Potent and selective human neuronal nitric oxide synthase inhibition by optimization of the 2-aminopyridine-based scaffold with a pyridine linker. J Med Chem. 2016;59(10):4913-4925. 10.1021/acs.jmedchem.6b00123

Wu Y, Yang Y, Ye S, Jiang Y. Structure of the gating ring from the human large-conductance Ca^{2+}-gated K^+ channel. Nature. 2010;466:393–397. 10.1038/nature09252

Yang F, Ling S, Zhou Y, Zhang Y, Lv P, Liu S, et al. Different conformational responses of the β_2-adrenergic receptor-Gs complex upon binding of the partial agonist salbutamol or the full agonist isoprenaline. Natl Sci Rev. 2021;8(9):nwaa284. 10.1093/nsr/nwaa284

Zhang H, Kong Q, Wang J, Jiang Y, Hua H. Complex roles of cAMP–PKA–CREB signaling in cancer. Exp Hematol Oncol. 2020;9:1-13. 10.1186/s40164-020-00159-7
